# A low-cost solution for documenting distribution and abundance of endangered marine fauna and impacts from fisheries

**DOI:** 10.1371/journal.pone.0190021

**Published:** 2017-12-28

**Authors:** Nicolas J. Pilcher, Kanjana Adulyanukosol, Himansu Das, Patricia Davis, Ellen Hines, Donna Kwan, Helene Marsh, Louisa Ponnampalam, John Reynolds

**Affiliations:** 1 Marine Research Foundation, Kota Kinabalu, Sabah, Malaysia; 2 Upper Gulf of Thailand Marine and Coastal Resource Research and Development Center, Samut Sakhon, Thailand; 3 Environment Agency Abu Dhabi, Abu Dhabi, United Arab Emirates; 4 Community Centered Conservation, London, United Kingdom; 5 Romberg Tiburon Center for Environmental Studies, Department of Geography & Environment, San Francisco State University, San Francisco, California, United States of America; 6 Convention on Migratory Species Office, Abu Dhabi, United Arab Emirates; 7 James Cook University, Townsville, Queensland, Australia; 8 The MareCet Research Organization, Shah Alam, Malaysia; 9 Mote Marine Laboratory, Sarasota, Florida, United States of America; Havforskningsinstituttet, NORWAY

## Abstract

Fisheries bycatch is a widespread and serious issue that leads to declines of many important and threatened marine species. However, documenting the distribution, abundance, population trends and threats to sparse populations of marine species is often beyond the capacity of developing countries because such work is complex, time consuming and often extremely expensive. We have developed a flexible tool to document spatial distribution and population trends for dugongs and other marine species in the form of an interview questionnaire supported by a structured data upload sheet and a comprehensive project manual. Recognising the effort invested in getting interviewers to remote locations, the questionnaire is comprehensive, but low cost. The questionnaire has already been deployed in 18 countries across the Indo-Pacific region. Project teams spent an average of USD 5,000 per country and obtained large data sets on dugong distribution, trends, catch and bycatch, and threat overlaps. Findings indicated that >50% of respondents had never seen dugongs and that 20% had seen a single dugong in their lifetimes despite living and fishing in areas of known or suspected dugong habitat, suggesting that dugongs occurred in low numbers. Only 3% of respondents had seen mother and calf pairs, indicative of low reproductive output. Dugong hunting was still common in several countries. Gillnets and hook and line were the most common fishing gears, with the greatest mortality caused by gillnets. The questionnaire has also been used to study manatees in the Caribbean, coastal cetaceans along the eastern Gulf of Thailand and western Peninsular Malaysia, and river dolphins in Peru. This questionnaire is a powerful tool for studying distribution and relative abundance for marine species and fishery pressures, and determining potential conservation hotspot areas. We provide the questionnaire and supporting documents for open-access use by the scientific and conservation communities.

## Introduction

Entanglement in fishing gear is the most serious threat to marine mammals because they are long-lived, slow-reproducing species for which survivorship, particularly in adults, is a very influential demographic parameter [1,2]. For the same reason, incidental mortality is also a threat to several other non-target species of megafauna such as sharks and sea turtles [3]. Species caught incidentally are often referred to as bycatch, although this term is often misleading because it may be inferred that the animals so-caught are discarded. In developing countries, many species of marine wildlife caught incidentally are retained for food [2]. All these non-target species are accidentally caught in both commercial and artisanal fisheries, where the impact of artisanal or traditional gillnet fisheries is believed to be particularly significant because of the large number of vessels operating in nearshore waters. Pauly [4] estimates that artisanal fishers comprise seven out of eight of the world’s fishers. Unlike industrial or large-scale commercial fisheries, there is limited regulation of artisanal fisheries and the use of gillnets, which is the gear type that poses the greatest threat to marine megafauna [3,5], is prevalent.

These small-scale fisheries occur primarily in developing nations with limited capacity and resources to obtain data, and where fisheries management is limited or non-existent, hindering comprehensive evaluations of the impacts of incidental capture of marine wildlife. In addition, the large number and small size of the boats makes observer programs prohibitively expensive and logistically challenging. Together, poor governance, poverty, and a lack of resources for local fisheries managers also preclude the use of modern technology for fisheries and bycatch assessments, such as video cameras, which are increasingly used in commercial fisheries in high income countries (http://www.nmfs.noaa.gov/stories/2015/09/fishermen_cameras_tracking_bycatch.html).

Measuring this effort and the impact that small-scale artisanal fisheries have on non-target species in a standardised and systematic manner has been a longstanding challenge. This knowledge gap is a major challenge to the effective conservation and management of threatened species [6].

There is increasing appreciation within the conservation community of the need to engage with fishers directly to address knowledge gaps and to involve them in developing solutions to management problems [7,8]. Collaboration with local fishing communities and effective collection and synthesis of information on capture rates (of target and non-target species) are seen as key requisites in the development of effective management measures [7,8]. Interview surveys are considered to be one of the most inexpensive and practical techniques to derive fishery data [9,10,11]. Many researchers now use interviews to quantify fishery effort and gather information on both targeted and incidental catch [3,6,12]. The use of local and traditional knowledge derived via these interview processes has been shown to be relatively accurate for fishery bycatch studies [6], large-scale benthic surveys [13], and stock assessments [14], and is a cost-effective approach to data collection. This approach is also useful for detecting changes over time [15], providing valuable insights for scientists, conservation managers, and policymakers [16,17].

Moore et al. [6] developed a short questionnaire to record the two primary types of information needed to quantify and spatially characterise incidental catch in fisheries in developing countries: a measure of fishing effort and a measure of incidental catch (bycatch)[12]. The Rapid Bycatch Assessment protocols (http://bycatch.env.duke.edu/) developed and field tested by Moore et al. [6] provided valuable information from seven countries spanning West and East Africa, Asia and the Caribbean. Even though a lot was learnt from these surveys, Moore et al. [6] acknowledged several drawbacks in their own study design and results: inadequate descriptions of interview methodology and lack of standardised interview protocols meant that data reliability was difficult to assess and results across studies were not comparable. Other key limitations to the study included inconsistency in application and sampling design (limited training and support materials); lack of integration with social sciences (surveys were designed by natural science practitioners); limitations on information returns (mostly short 5-minute interviews and occasional 30-minute surveys); and data reliability. Moore at al. [6] acknowledged these drawbacks, and a second set of Rapid Bycatch Assessment questionnaires was developed in response to extensive analysis of these limitations. These revised surveys were not subsequently widely used, primarily due to funding limitations (R. Lewison, pers. comm., 31 Mar 2016).

Dugongs (*Dugong dugon*) are seagrass-dependent medium-sized marine mammals found in the coastal and island waters of more than 38 Indo-west Pacific tropical and sub-tropical countries, only eight of which were classified in 2015 as countries with a Very High Human Development Index by the United Nations Development Programme (http://hdr.undp.org/en/composite/HDI). Throughout much of their range, dugong populations are mostly small and fragmented and so the mortality of even a few animals per year can have a serious impact on the long-term viability of a local population. In many regions, the incidental catch of a dugong is a relatively rare event for an individual fisher, and is often considered inconsequential by fisheries managers [2], but these mortality events can have profound impacts on small populations. In some areas, the problem is exacerbated because the targets of certain gillnetting operations are extremely valuable, e.g. shark fin [2].

Population declines have been reported from much of the dugong’s range [2,18]. The International Union for the Conservation of Nature (IUCN) classifies dugongs as Vulnerable to extinction on a global scale [19,20]; most populations are small and dwindling, but the IUCN assessment is buffered by large populations in Northern Australia and the Arabian Gulf [2]. Many dugong populations would likely be considered as Endangered or Critically Endangered if they were assessed at regional scales, and in some countries these populations are listed as Endangered under National legislation [2].

In this paper, we describe the development and use of a questionnaire survey tool deployed successfully by projects affiliated with the UNEP-CMS Dugong MoU Secretariat in 18 countries spanning four key regions across the Indo-Pacific. The use of the questionnaire resulted in 6,153 data sets on targeted and incidental catch across the majority of the dugong’s range. The surveys were administered by local agencies in dugong range states in areas previously recorded as dugong and seagrass habitats by local research and conservation agencies. We also provide several concrete examples of the survey outcomes that highlight the value of the tool, and on the robustness of the questionnaire when compared with independent data collected from direct observations. The questionnaire can be easily adapted and modified for different species, and we provide the questionnaire and supporting documents for open-access use by the scientific and conservation communities.

## Description and design of the Dugong Catch & Bycatch Questionnaire

The Dugong Catch & Bycatch Questionnaire (S1 Appendix) presented herein was based on the follow-up version of the Rapid Bycatch Assessment survey, streamlined to meet ethical considerations and structure protocols such as those in use at the Phuket Marine Biological Center (Thailand), San Francisco State University (USA) and James Cook University (Australia). The questionnaire was prompted by the urgent need for spatial, trend and abundance information on dugongs and the impacts of small-scale fisheries, data that would enable signatories to the UNEP-CMS Dugong Memorandum of Understanding to more efficiently address conservation challenges. Key requisites for the questionnaire were that it be widely applicable across regions and issues, scientifically sound and robust, and culture-sensitive. This questionnaire expands and improves on earlier rapid bycatch assessment efforts primarily by:

1. *Involving social scientists in the design of the survey*. The involvement of social scientists in the survey design allowed us to address concerns over interview processes, question design, and ethics, as addressed in detail by White et al. [21] and Lowe et al. [22], among others;
2. *Expanding the survey to include questions on multiple species and on both factual information and perceptions of the interviewee*. The survey was designed to balance the conflicting demands of information value and respondent fatigue. Whereas short surveys may reduce non-response rates [21], such surveys often limit the amount of information that may be derived after investing substantial resources into travel and accessing remote communities. Longer interviews can help maximise the accuracy of the information obtained via the interview [23]. Our questionnaire has 49 questions related to the interviewee, dugongs, and fisheries, an additional 51 optional questions on turtles and other marine mammals, and 6 questions related to response quality. Completing 106 questions is time consuming, and may result in fatigue for both interviewer and interviewee. Nonetheless, interviewers learn considerably as surveys progress and are able to shorten the interview process [24]. We found that the duration of each interview dropped substantially as interviewers became more familiar with the questions, the logical order of the questionnaire, and the response options. Interviews conducted by novice interviewers lasted up to one hour, but experienced interviewers could cover the same questions in 30–40 minutes;
3. *The inclusion of an ‘*I don’t know’ *response to minimise misinformation*. The ‘I don't know’ option allowed respondents to state that they had no opinion or knowledge to respond to a particular question (respondents might legitimately not know the answer to a factual question);
4. *Field-testing and revision prior to widespread implementation*. The questionnaire was field tested in Northern Australia, Papua New Guinea, the United Arab Emirates and Malaysia by different researchers who provided feedback in the design and ease of implementation;
5. *A series of questions on data integrity and reliability* (which could assist in justifying removal of questionable data sets). Whereas there has been widespread concern over the accuracy and validity of interview-based bycatch data gathering [23], there is evidence that the process can result in relatively accurate findings [25,26], at least at the broad-scale [6];
6. *An ethics statement on each questionnaire*, in keeping with University and other ethics board requirements for major social studies such as face-to-face interviews;
7. *Clear and transparent*, *simplified data analysis via the provision of an Excel data upload sheet* (provided with the survey; S2 Appendix); and
8. *The provision of a comprehensive training manual for each user* (provided alongside the survey; S3 Appendix);
9. *The inclusion of a spatial component that can be linked to geographic information systems (GIS) software via the widely accessible Google Earth™ platform*. This process can be used to depict local knowledge and explore relationships between resources and threats, and identify hotspot areas via GIS platforms [27]. These maps can be used by managers to identify areas where there are substantial overlaps in fishing effort and species of concern (e.g. dugongs) or aid in the designation of marine protected areas [28,29].

The dugong component of the questionnaire contains 49 questions: 16 relate to the informant and his/her livelihood; 22 to dugong catch and bycatch; eight to perceptions the informant may have on dugong importance and trends, and three relating to the fishery and gears used. An additional optional 26 questions relate to sea turtles and another 25 to other marine mammals. A final six questions are questions internal to the survey and relate to the interviewer’s assessment of interviewee confidence, knowledge and accuracy. These questions are provided to enable potential future assessments of the robustness of the data. Several questions cross check information relayed by the interviewee to strengthen data integrity. The interviewee is invited to record his/her fauna sightings and fishing areas on clean maps during the deployment of the questionnaire, and the questionnaire includes a data table to record the details of each of these sightings. The spatial component is one of the key strengths of the process as it captures locations of sightings, fishing pressure and seagrass distribution. Maps and sighting tables are linked to each questionnaire as follows: Each questionnaire is assigned a unique identification (ID) number, in sequential order with a country ISO code prefix. Each unique ID is keyed in to the Excel spreadsheet for each record, and each map is similarly labeled. Data uploaded into Google Earth™ are also linked to the unique ID code at the time when point and polygon data are labeled. In this manner the respondent’s personal, catch and bycatch, effort, trend and spatial information are all linked providing data transparency and traceability.

**Fig 1:**
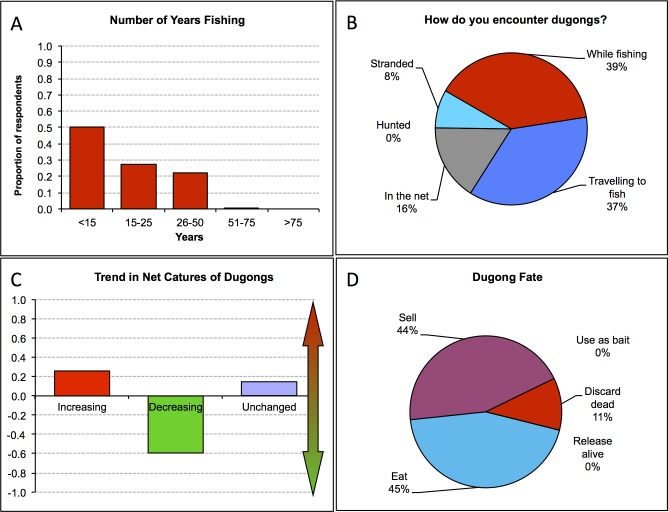
Samples of some of the standardised graphical data outputs at different spatial scales: (a) proportion of respondents across 18 countries that had been fishing for various numbers of years; (b) proportion of respondents across 18 countries who encountered dugongs in different ways; (c) trends in dugong captures in Sabah, Malaysia; and (d) fate of dugongs after being caught in nets in Malaysia.

A standardised Excel spreadsheet (S2 Appendix) was developed into which data may be uploaded, with locked fields controlled via filters and validation to minimise data entry errors. Locked formula cells process the data in real time and construct 27 different graphic and numerical chart outputs in a standardised format, so that data may be similarly interpreted from location to location. The formulas do not extend to statistical functions, but rather calculate proportional contributions of categories for simple data visualisation related to respondent demographics, fishing vessel and gear types, dugong numbers and trends, and perceptions of changes and importance of dugongs by the respondents (Fig 1). Users are unable to edit the worksheet of modify the graphs, but are able to copy their data into a new file and analyse separately and more thoroughly should they wish.

**Fig 2:**
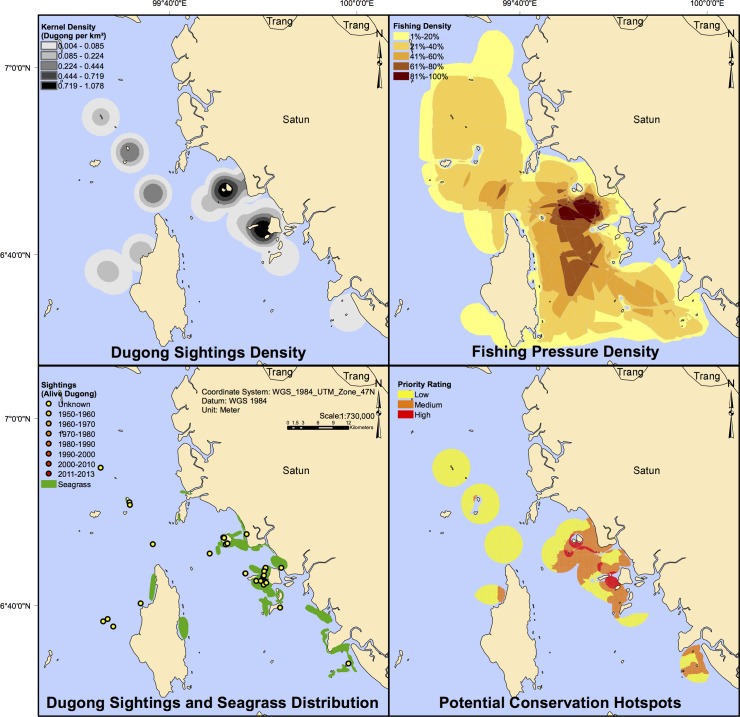
Dugong and fishery density analysis, sightings and seagrass distribution, and priority area identification for Satun Province along the South Andaman Sea Coast, Thailand. Source: [30].

In addition to updating information on dugong mortality and trends, spatial distribution, and a determination of where the number of dugongs was low and the threats to their existence was high, the deployment of the questionnaire across many CMS dugong range States has enabled a wide-scale spatial analysis of areas where small-scale fisheries and dugongs overlap, and the identification of potential ‘hotspot’ areas where relevant resource management agencies may wish to focus initial conservation and management efforts (Fig 2). This updated understanding of the spatial distribution of dugongs, threats and their habitats is an important tool for the management of marine resources [29].

**Fig 3:**
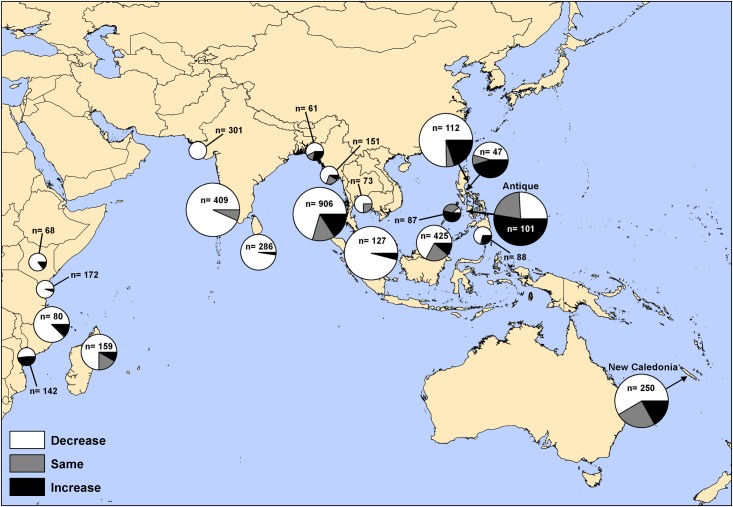
An example of regional-level GIS outputs derived via the questionnaire data: Collated fisher responses to trends in dugong captures in fishing gear across all recorded years, scaled by numbers of dugongs encountered at each location: small pie charts <50 dugongs reported; medium pie charts 50–100 dugongs reported; large pie charts >100 dugongs reported; dugong captures increasing (black segments), decreasing (white segments) or remaining the same (grey segments). Note that reports of dugong sightings could include multiple reports of the same individual. Source: [30].

Additional GIS-based hotspot analyses using raw data derived by the questionnaire highlighted how areas of heightened interest may be prioritised for additional research, and allowed us to examine the trends in dugong populations at a greater regional level than was previously possible, for instance by analysing trends in dugong captures over time, such as that provided in Fig 3. This graphic illustrates that trends in net captures are increasing at an alarming rate in Antique (Philippines) for instance, suggesting this location is urgently in need of programmes to address net captures, whereas net captures are generally decreasing in New Caledonia. These findings are context specific and need to be interpreted with caution: An increasing trend in net captures is of concern unless there is independent evidence that the population is increasing. A decreasing trend in reports of dugongs being captured in nets could indicate a (good) decrease in captures, or a (bad) decrease in dugong abundance.

A Project Manual (S3 Appendix) was assembled to explain the project rationale and introduce the Dugong Catch & Bycatch Questionnaire and help users implement the process. The manual introduces topics such as interview methods and techniques, data integrity, survey design effort and efficiency, stratified and random sampling, field data collection and control, and how to link graphics to table data and survey numbers. Other chapters address uploading graphics and spatial data and creating and exporting Google Earth layers to a commercial or open-source GIS software package, and basic GIS analyses once all data are uploaded. The questionnaire and both the Excel sheet and the Project Manual are available as supplementary material.

Finally, the survey design addresses the ethical considerations of administering the questionnaire and the use of the data. Administration of questionnaires such as these is typically governed by ethics boards at Universities, NGOs and government institutions, but we recognised from the outset that the questionnaire was going to be implemented in developing countries by government agencies and NGOs that may not have ethics boards of their own. Cognizant of human rights and ethics protocols, we developed an Introductory/Ethics Statement to reflect the ethics board requirements of Universities in the US and Australia. This Introductory statement also adheres to the spirit of Free, Prior and Informed Consent (FPIC) of the United Nations Permanent Forum on Indigenous Issues. The Introductory statement to every questionnaire is on the initial page of the questionnaire.

## Examples of key findings and survey robustness

The questionnaire was used by 18 CMS range States resulting in 6,153 respondents from four regions: East Africa (Kenya, Madagascar, Mozambique and Tanzania), South Asia (Sri Lanka, Bangladesh and India), Southeast Asia (Cambodia, Thailand, Vietnam, Myanmar, Malaysia and Philippines), and the Pacific Islands (Palau, Papua New Guinea, Solomon Islands, New Caledonia, and Vanuatu). We provide here a subset of the results from this process to demonstrate the value of the questionnaire tool.

The questionnaire was deliberately targeted at fishers because they generally have the greatest opportunity to interact with dugongs. Hence, 89.1% of respondents were fishers, followed by those in tourism-related occupations (1.1%), retirees (1.1%) and the aviation sector (0.9%), There were a total of 67 ‘Other’ employment records, including farmer (20), gardener (8), driver (6), security guard (5), mechanic (4), student (3), waitress (2), cleaning lady (2), and others. Given the large proportion of fishery sector respondents, it was not surprising that 96% of all respondents were male.

Just over half of all respondents indicated they had never seen a dugong (51.4%) presumably reflecting the dugong’s low numbers across the four geographic areas. In a subsequent question which was designed to assess response reliability, a similar 51.2% of respondents mirrored this finding (Table 1). The similarity across these responses provides a measure of reassurance of the questionnaire findings. About a fifth of respondents indicated they had only seen one dugong in their lifetime (20.1%); and only a very low proportion indicated seeing them every year (1.9%; Table 1). Among all respondents for which data are available, only 3% reported seeing dugong calves, a much lower percentage than generally seen, for instance, during dugong aerial surveys in Australia [31].

**Table 1:** Sighting frequency of dugongs across all respondents. Given the temporal spread of questionnaire deployment, the ‘last year’ typically referred to 12-month periods between 2011 and 2012.

In the last year		In a lifetime	
Every day	0.6%	Every year	1.9%
Every week	1.2%	Frequently	6.9%
Every month	5.7%	A few times	20.0%
Several times	48.7%	Only once	20.1%
Only once	43.8%	Never	51.2%

**Fig 4:**
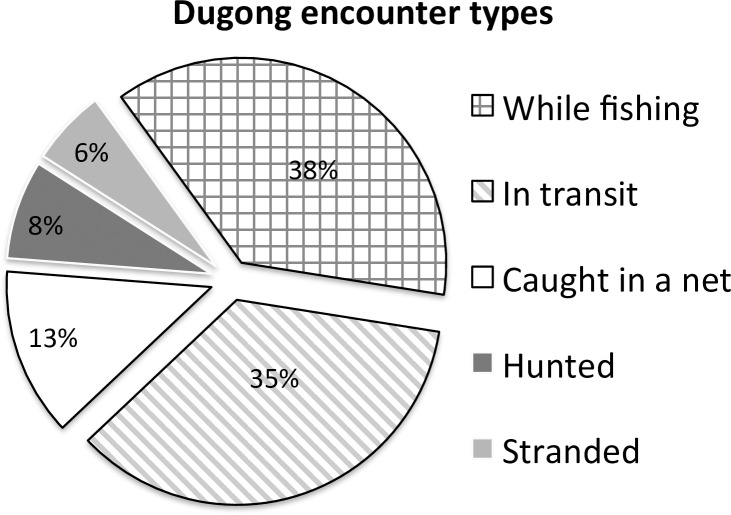
Types of dugong encounters reported by 2,611 respondents across all countries.

The questionnaire also addressed direct and indirect capture by asking how dugongs were ‘encountered’. The breakdown of reported encounter types was reflective of the questionnaire’s focus on fishers, and notably highlighted that dugongs continued to be hunted (7.8% or respondents; Fig 4). The greatest number of respondents reporting hunting of dugongs in India (where 44% of all hunting was recorded), Tanzania (18% of all hunting records) and the Solomon Islands (15%). A subset of 37% of respondents was not asked about how dugongs were encountered, as interviewers sometimes found this was a delicate subject to broach given the ban on killing of dugongs in most range states [2]).

**Fig 5:**
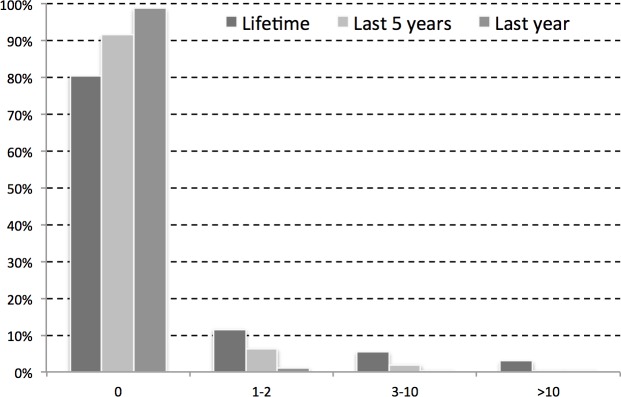
Dugong capture records from 3,503 respondents.

Respondents were asked to report on catches in the last year, the last five years, and over their lifetimes. The overall number of dugong catches appeared to have declined over the time, a result that likely reflects the declining number of dugongs, rather than any effort to reduce interactions (Fig 5). The high proportion of respondents (~80–90%) reporting zero captures was not a conflict with the lower proportion of respondents reporting zero sightings (Table 1) because not all sightings result in captures.

**Fig 6:**
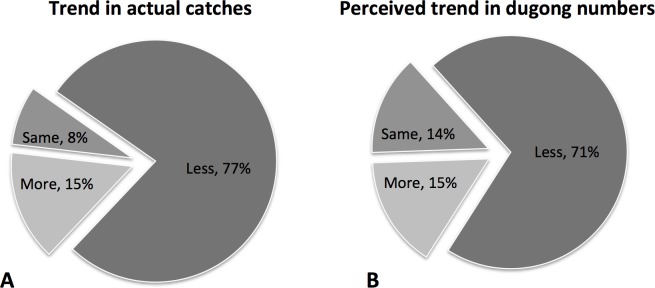
Trend in reported catches of dugongs in fishing gear (A) and perceived trends in dugong population size as reported by fishers (B).

Respondents were asked to indicate if the number of dugongs caught in fishing gears was increasing, decreasing or remaining the same. Mirroring the data on numbers of dugongs, ~77% of fishers reported a decrease in catches over time (Fig 6). Interestingly, a similar proportion of fishers also perceived that dugong populations were declining, highlighting the understanding fishers have for the environment and the species with which they interact (Fig 6). Respondents indicated that accidentally bycaught dugongs were more often released alive (52%), discarded dead (16%), eaten (16%), or sold or used as bait (11% respectively). We cannot assess the reliability of the high claims of live releases because respondents might not admit that a dugong drowned or was eaten, but we suggest this is likely an overestimate of dugong numbers reportedly caught and released alive.

The two most common fishing gear types used by respondents were gillnets (27%) and hook and line (25%; Table 2). By far the greatest proportion of dugongs was caught in gillnets (27%), with the smallest proportion coming from trawl fisheries (1%). Captures were fairly evenly split among other gear types. These findings further reaffirm a common concern regarding the impact of gillnets, but also suggests greater focus is needed on artisanal hook and line fisheries that impact dugongs.

**Table 2:** Proportional gear use and encounters of dugongs by gear type across all respondents. The proportional catch by gear type involves only those reports for which actual dugong captures were reported.

	Proportional gear use	Proportional dugong catch by gear type
Hook & Line	25%	15%
Bottom Longline	7%	12%
Longline	10%	16%
Trawler	8%	1%
Beach Seine	9%	14%
Purse Seine	13%	14%
Gillnet	27%	27%

**Fig 7:**
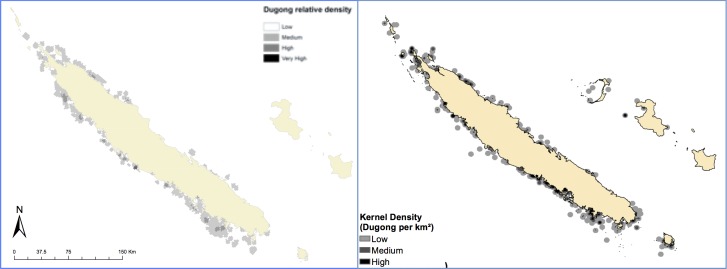
Dugong distribution in the nearshore waters of the main islands of New Caledonia as determined via aerial surveys (left) and the dugong questionnaire and fisher feedback on sightings (right). Data sources: [30,32].

Opportunistically, the timing of the deployment of the survey in New Caledonia allowed a comparison of the dugong density derived from the spatial component of the questionnaire with the results of actual sightings during aerial surveys (Fig 7). The aerial survey density map of dugong distribution was made based on calculations of direct aerial survey sampling effort corrected for sampling intensity variations between blocks and across surveys; and models dugong distribution and relative density using geostatistics [32]. The questionnaire density graphic was developed via a kernel density analysis of sightings indicated by fishers that were annotated manually on the maps. In New Caledonia the aerial survey dugong density categories were fixed quartiles, whereas the questionnaire data were processed as 90% home range and 50% core areas based on dugong sighting records. The questionnaire-derived map overlapped well with the aerial survey map when with regards to dugong presence, and indicated some areas of dugong presence where none were observed during aerial surveys. Whereas the density scales differed between the two analyses, there was a clear similarity between the data set derived via fisher interviews and the spatial component of the questionnaire (Fig 7, right) when compared with the actual data derived via sightings during aerial surveys (Fig 7, left). This result indicates that the spatial component of the questionnaire was able to provide reliable information on distribution of dugongs at a fraction of the cost of more expensive aerial surveys. Of value, the questionnaire data also identified dugong presence on the outer islands of New Caledonia, where aerial surveys were not conducted.

## Value and limitations

Despite several regional training workshops at which the questionnaire was introduced, some project partners implemented the questionnaire but did not return any of the spatial data, while others conducted independent analyses and only reported the summarised findings rather than the raw data sets. Some partners did not use the maps. Others used the maps but did not report the data. We suggest that careful attention to the instructions in the Manual are required, and where possible dedicated training for at least each project coordinator to enable a more thorough understanding of the various programme components and the types of information which can be derived by implementing the protocols outlined in the manual.

Given the nature of the questions and the variability in responses, we recognise there may be areas of underreporting [33], potential bias and respondent misinformation, and that the questionnaire does not provide absolute numbers, exact population abundance estimates or precise locations of fishing and dugong areas. Rather, the questionnaire provides a rapid, low cost solution to acquiring preliminary marine species and fishery data incorporating local and traditional knowledge that may be assimilated into conservation and management efforts [34,35, 36].

Notwithstanding the limitations on the administration of the survey questionnaire, the large number of respondents for which mostly-complete data sets were available provided a wealth of information on the endangered status of dugongs across major areas of the Indian and Pacific Oceans, and updated our knowledge of distribution, numbers, and trends.

The questionnaire has already been used to study several other species of marine mammals including manatees in the Caribbean, coastal cetaceans along the eastern Gulf of Thailand and western Peninsular Malaysia, as well as river dolphins in Peru, and has continued to be used by several project affiliates since its inception. The data also contributed to the design of the Dugong and Seagrass Conservation Project, a USD5.88 million Global Environment Facility (GEF) grant supporting 38 projects distributed across Indonesia, Madagascar, Malaysia, Mozambique, Timor Leste, Sri Lanka, the Solomon Islands and Vanuatu. The questionnaire has also been used to further the understanding of dugong distribution, trends and threats in Egypt (200 additional interviews completed), Indonesia (124), Malaysia (900), Mozambique (184), and Vanuatu (460). Given the value of these data relative to the cost of procurement, the UNEP-CMS Dugong MoU Secretariat intends to use the questionnaire at additional locations in new locations to develop pilot projects to provide incentives to fishing communities to manage fishing interactions with dugongs. The questionnaire also features in the Dugong and Seagrass electronic research toolkit developed to assist researchers in developing range states (www.conservation.tools/).

The spatial analysis component of the analysis protocol enabled the identification of ‘hotspot’ areas where dugongs and fisheries overlap. These data, along with the graphic outputs of the Excel sheet and the GIS analysis can be used to highlight priority areas for further detailed study and assessments. We provide these tools and simplified, anonymous data as supplementary material for the use of the scientific and conservation communities working not just on dugongs but also on other threatened marine megafauna.

## Supporting information

S1 Appendix UNEP- CMS Dugong Catch & Bycatch Questionnaire. (DOC)

S2 Appendix Dugong Catch & Bycatch Questionnaire data upload sheet. (XLSX)

S3 Appendix Dugong Catch & Bycatch Questionnaire project manual. (PDF)

## References

[1] Read AJ. The looming crisis: interactions between marine mammals and fisheries. J Mammal. 2008; 89, 541–548.

[2] Marsh H, O’Shea TJ, Reynolds JE III. The ecology and conservation of Sirenia: dugongs and manatees. Cambridge University Press; 2011.

[3] Lewison RL, Crowder LB, Read AJ, Freeman SA. Understanding impacts of fisheries by-catch on marine megafauna. Trends Ecol Evolut. 2004; 19: 598–604.

[4] Pauly D. Major trends in small-scale marine fisheries, with emphasis on developing countries, and some implications for the social sciences. MAST. 2006; 4(2), 7–22.

[5] Lewison RL, Soykan CU, Cox T, Peckham H, Pilcher NJ, LeBoeuf N, et al Ingredients for addressing the challenges of fisheries bycatch. Bull Mar Sci. 2011; 87(2): 235–250.

[6] Moore JE, Cox TM, Lewison RL, Read AJ, Bjorkland R, McDonald SL, et al An interview–based approach to assess marine mammal and sea turtle captures in artisanal fisheries. Biol Cons. 2010; 143: 795–805.

[7] Jentoft S. The community: a missing link of fisheries management. Mar Poll Bull. 2000; 24: 53–9.

[8] Chuenpagdee R, Fraga J, Euán-Avila JI. Progressing toward comanagement through participatory research. Soc Nat Res. 2004; 17: 147–61.

[9] Aragones LV, Jefferson TA, Marsh TA. Marine mammal survey techniques applicable in developing countries. Asian Mar Biol. 1997; 14: 15–39.

[10] Jones JPG, Andriamarovololona MM, Hockley N, Gibbons JM, Milner-Gulland EJ. Testing the use of interviews as a tool for monitoring trends in the harvesting of wild species. J Appl Ecol. 2008; 45: 1205–1212.

[11] Ortega-Argueta A, Hines E, Calvimontes J. Using Interviews in Sirenian research In Hines E, Reynolds J, Mignucci-Giannoni A, Aragones LV, Marmontel M, editors. Sirenian Conservation: Issues and Strategies in Developing Countries. Gainesville The University Press of Florida; 2012 pp. 109–115.

[12] Hall M, Nakano H, Clarke S, Thomas S, Molloy J, Peckham SH, et al Working with fishers to reduce by-catches In Kennelly S, editor. By-catch reduction in the world’s fisheries. Dordrecht: Springer; 2007 pp. 235–288.

[13] Teixeira JB, Martins AS, Pinheiro HT, Secchin NA, Leão de Moura R. Bastos AC. Traditional Ecological Knowledge and the mapping of benthic marine habitats. J Environ Manag. 2013; 115: 241–250. 10.1016/j.jenvman.2012.11.020 23262411

[14] Fairclough DV, Brown JI, Carlish BM, Crisafulli BM, Key IS. Breathing life into fisheries stock assessment with citizen science. Sci Rep. 2014; 4: 7249 doi: 10.1038/srep07249 25431103 10.1038/srep07249 PMC5384193

[15] Tesfamichael DT, Pitcher J, Pauly D. Assessing changes in fisheries using fishers’ knowledge to generate long time series of catch rates: a case study from the Red Sea. Ecol Soc. 2014; 19(1): 18.

[16] Bart D. Integrating local ecological knowledge and manipulative experiments to find the causes of environmental change. Front Ecol Environ. 2006; 4: 541–546.

[17] Stave J, Oba G, Nordal I, Stenseth NC. Traditional ecological knowledge of a riverine forest in Turkana, Kenya: implications for research and management. Biodivers Conserv. 2007; 16: 1471–1489.

[18] Marsh H, Penrose H, Eros C, Hugues J, 2002 Dugong: Status Reports and Action Plans for Countries and Territories. United Nations Environment Programme World Conservation Monitoring Centre, Cambridge: UNEP/DEWA/RS.02-1; 2002.

[19] IUCN Red List of Threatened Species. Version 2015–4. Available from <www.iucnredlist.org>. Downloaded on 15 May 2016.

[20] Marsh H, Sobtzick, 2015 S. Dugong dugon. The IUCN Red List of Threatened Species 2015. Available from http://dx.doi.org/10.2305/IUCN.UK.2015-4.RLTS.T6909A43792211.en.

[21] White PCL, Jennings NV, Renwick AR, Barker NHL. Questionnaires in ecology: a review of past use and recommendations for best practice. J Appl Ecol. 2005; 42: 421–430.

[22] Lowe P, Whitman G & Phillipson J. Ecology and the social sciences. J App Ecol. 2009; 46: 297–305.

[23] Huntington HP. Using traditional ecological knowledge in science: methods and applications. Ecol Appl. 2000; 10: 1270–1274.

[24] M & Stöhr T. Guidelines for the use of household interview duration analysis in CAPI survey management; 2012. Kiel Institute for the World Economy, Germany. Kiel Working Paper No. 1779.

[25] Lozano M, Baro J, García T, Frías A, Rey A, Báez JC. Loggerhead sea turtle bycatch data in artisanal fisheries within a marine protected area: fishermen surveys versus scientific observations. Anim Biod Conserv. 2011; 34(1): 31–34.

[26] Sobtzick JRS, Valduga AT, Restello RM, Cardoso RI, Hepp LU, Siqueira AB. Local ecological knowledge as a complementary basis for the management of water resources. Ethnobio Conserv. 2013; 2: 10.

[27] Close CH, Hall GB. A GIS based protocol for the collection and use of local knowledge in fisheries management planning. J Environ Manag. 2006; 78:341–352. 10.1016/j.jenvman.2005.04.027 16115723

[28] Caddy JF, Carrocci F. The spatial allocation of fishing intensity by port-based inshore fleets: A GIS application. ICES J Marine Sci. 1999; 56: 388–403.

[29] Valavanis VD, Georgakarakos S, Kapantagakis A, Palialexis A, Katara I. A GIS environmental modeling approach to essential fish habitat designation. Ecol Model. 2004; 178: 417–427.

[30] Pilcher NJ, Williams J, Hopkins G, Hess D, Jaouen L. UNEP-CMS Questionnaire Survey: Assessment of dugong distribution and interactions with small-scale fisheries Abu Dhabi, UNEP-CMS Office United Arab Emirates; 2017; 87pp.

[31] Fuentes MMPB, Beatty B, Delean S, Grayson J, Lavender S, Logan M, et al Spatial and temporal variation in the effects of climatic variables on dugong calf production. PLoS One 2016; PONE-D-15-52097R1 10.1371/journal.pone.0155675 PMC4927176 27355367

[32] Cleguer C, Grech A, Garrigue C, Marsh H. Spatial mismatch between marine protected areas and dugongs in New Caledonia. Biol Conserv. 2015; 184, 154–162.

[33] Silver JS & Campbell LM. Fisher participation in research: Dilemmas with the use of fisher knowledge. Ocean Coast Manag. 2005; 48, 721–741.

[34] Ruddle K. Local knowledge in the future management of inshore tropical marine resources and environments. Nat Resour. 1994; 30(1): 28–37.

[35] Gadgil M, Berkes F, Folke C. Indigenous knowledge for biodiversity conservation. Ambio 1993; 22: 151–156. 10.1007/s13280-020-01478-7 PMC8035379 33566330

[36] Drew JA, Henne AP. Conservation biology and traditional ecological knowledge: Integrating academic disciplines for better conservation practice. Ecol Soc. 2006; 11(2): 34.

