# Supplementary material for: A low-cost solution for documenting distribution and abundance of endangered marine fauna and impacts from fisheries
Source: PLoS One. 2017 Dec 28;12(12):e0190021. doi: 10.1371/journal.pone.0190021 (PMC5746211; doi:10.1371/journal.pone.0190021)
Supplement: S1 Appendix — (DOC) [file pone.0190021.s001.doc]

**Standardised Dugong Catch / Bycatch Questionnaire**

Interviewer Name: Date: Data Sheet Serial Number:

Town: Province:

**introducTIon statement**

Note: Reading this statement to the interviewee is compulsory. It ensures all interviews are treated equally.

My name is . I work for a project run by the , which is an organization based in (*insert location*) which is supporting research to protect the ocean for fishers and wildlife. The goal of this project is to learn more about capture of dugongs and any other marine wildlife in coastal fisheries of (*insert location*). We would like to ask you some questions about wildlife you have seen, what fishing gear you use (if any), where you fish, and questions like that. We have maps and pictures that can be used to help answer some of the questions. The questions will take between 30 to 45 minutes to complete. Information from our research could be used to help reduce the capture of dugongs and other marine mammals and sea turtles, maybe through direct community support for our goals, or possibly through more effective regulations and enforcement. **Your participation in this survey is voluntary and confidential. We will not record your name or any personal information you share with us unless this is ok with you. Individual answers will be collated and reported on as a group to provide a general idea of current status, and we will absolutely not share your individual answers to anyone outside of the research team. You do not have to answer questions you do not want to. THANK YOU FOR YOUR HELP !**

**Interviewee Background**

Note: Please tick the boxes to the left of any questions not asked.

1. Name:

2. Age: Gender: Male  Female

3. Have you previously participated in interviews related to:

Fishing  Marine Mammals  MPAs  Ecotourism  Sea Turtles  Other  None

When did you participate?

Describe:

4. What is your main occupation?

Fishing  Tour Guide  Boat Captain / Crew  Air Services  Retired

Other  Please describe:

5. For how many years has this been your occupation?

6. Do you have a fishing background? Yes  No

7. Were your parents fishers? Yes  No  Grandparents? Yes  No

8. Is fishing the main way you earn a living? Yes  No

9. Is fishing the only way you earn a living? Yes  No

(if no) What is (or are) your other occupation(s)?

10. Which months do you normally fish (out of the last 12)?

(*if seasonal, indicate season start and end*)

11. How many days each week do you fish? days (low season) days (peak season)

12. What is your position on the boat? The captain  A crew member  We have no fixed positions

I do not work on a boat  (*skip next questions if person does not work on a boat*)

13. How many fishers, including yourself, work on the boat?

14. How long is the boat?

(*Note to* *interviewer: convert and provide answer in meters*)

15. Is the boat motorized? Yes  No  (if yes) Inboard  Outboard

16. What is the horsepower of the motor?

**Dugong catch / bycatch**

17. Have you ever personally seen a dugong in our waters? Yes  No

Do you have another name for it?

18. Tell me about the difference between a dugong and a dolphin?

19. How long do you think a dugong lives? Don’t know

20. How do you get to see dugongs? Seen while fishing  Seen while travelling to fishing areas

Accidentally caught in nets  Hunted  Stranded on the beach  Other

Where do you normally fish? (*Note to interviewer: Complete attached table and mark all locations on maps*)

21. How frequently have you seen dugongs? Never  Once in my life  Only a few times in my life

Frequently  Every year for the last five years

In the last year, only once  several times  every month  every week  every day .

22. In what month(s) do you see dugongs? (*indicate months when seasons are used*):

23. When was the last time you saw a dugong? (*if long time ago note the year*)

24. Do you know of any areas where dugongs regularly occur? Yes  No

(*Note to interviewer:* Regular *means certain times of year when they are always found*)

Where are these special dugong areas? (*Indicate on map*)

25. Do these dugong areas change over time? Yes  No  Don’t Know

26. How many dugongs do you think might live in these areas? 1  <10  >10  Don’t Know

27. Have you ever seen dugong calves / babies? Yes  No  When? (*what month(s)?*)

Where did you see them? (*ask interviewee to* *show on maps*)

28. Do people from other villages / communities catch dugongs? Yes  No  Don’t Know

(*if yes*) How many (people)? What village?

Any other details?

Is the catch accidental or on purpose? Accidental  On purpose  Both

29. Do you have any dugong specialists or catchers in your village? Yes  No  How many?

30. Have people in your village / community ever caught dugongs? Yes  No  Don’t Know

(*if yes*) How many (people)? For how long? Any details?

Is the catch accidental or on purpose? Accidental  On purpose  Both

31. Did you personally catch any dugongs in the last year? (*accidentally or hunted*) Yes  No

(*if yes*) How many in the last year? 1-2  ≤10  >10  Any details? (*if available*):

Was this is a typical number to catch in a year? Yes  No

(*if no*) Was it higher or lower than usual? Higher  Lower

Was the catch accidental or was it something you were fishing for? Accidental  Hunted  Both

32. Did you catch any in the last five years? 0  1-2  ≤10  >10  Specifics (*if available*):

How many in your life? 0  1-2  ≤10  >10  Specifics (*if available*):

33. How did you catch them? Harpoon  Nets  Other  Please describe:

When did you catch them? (*what month(s)?*) Where? (*ask interviewee to* *show on maps*)

34. Compared to when you started fishing, are there more , less , or the same number of dugongs

hunted / captured in fishing gear? Don’t Know  (*Note: this is based on actual numbers, not perception*)

(if more or less) Why do you think this?

35. What do you (or would you) do with a dugong if you caught one on purpose?

Eat  Sell  As Bait  Other Use : *(Note: do not lead interviewee)*

36. What do you (or would you) do with a dugong if you caught one accidentally?

Discard (*dead*)  Release (*alive*)  Eat  Sell  As Bait  Other Use :

37. Have you ever found  or heard of  dugongs stranded on the shore? Yes  No  (*explain*)

Or have you ever found  or heard of  dugongs dead in our waters? Yes  No

Or have you ever found  or heard of  dugongs with cut marks on their backs? Yes  No  (*explain*)

(if yes) Where, when and how many? (*ask interviewee to* *show on maps*)?

What happened to the animal(s)?

38. What would you do if you found a stranded dugong?

**Perceptions**

39. Compared to when you started fishing, do you think there are more dugongs , less , or the same

number of dugongs ? I don’t know

(if more or less) Why do you think this?

(*Note to* *interviewer: Try to determine what other impacts may be driving the trend*)

40. Do you think there will always be dugongs in the sea? Yes  No  Don’t Know

(if yes or no) Why?

41. Do you think having dugongs around is important? Yes  No  Don’t know  Why?

42. Do you know what seagrass areas are? Yes  No  (*Note to* *Interviewer: show graphics*)

Are there any seagrass areas around here? Yes  No  Don’t know  Where?

(*Note to* *Interviewer: mark on maps*)

Do you fish in these seagrass areas? Yes  No

Are these seagrass areas important for anything else? Yes  No  Why?

43. Is it illegal to intentionally kill a dugong Yes  No  Don’t know

What about by accident (maybe caught in a net unintentionally)? Yes  No  Don’t know

Would you report an accidental mortality to the authorities? Yes  No

Details (if given)?

44. Are any areas routinely / periodically patrolled? Frequently  Infrequently  Never  Don’t know

45. If yes, are penalties ever imposed? Frequently  Infrequently  Never  Don’t know

46. Are there any local customs, beliefs, legends or rituals or stories related to dugongs? Yes  No

(if yes) Please describe:

Where / from whom did you hear this?

Any additional stories / incidents you wish to report:

**Fishery Information**

*Note to interviewer: Respondent should answer these questions to describe his/her individual experience, not that of their community. Use illustrations to assist where necessary.*

*Habitat Codes: (D) Deep Water; (C) Coral; (S) Seagrass; (F) Fine Sediments; (M) Mangroves; (R) Rocks;*

*(E) Estuaries; (U) Unknown*

47. What type of fishing gear do you use? (*Indicate what months*)

Gill or trammel nets Only  Mostly  Sometimes  Season:

Habitat: Target:

Do you tend the nets when they are in the water? Yes  No

How long do you leave the nets in the water? hours

Do you fish during the day  or night ? Both ?

What is the position of the gear? Surface  Mid-water  Bottom

Full water depth  (*normally in shallow waters*)

Describe the net: Length Depth Mesh size

Longline Only  Mostly  Sometimes  Season:

(*many hooks*) Habitat: Target:

Bottom longline Only  Mostly  Sometimes  Season:

(*many hooks set at depth*) Habitat: Target:

Hook and line Only  Mostly  Sometimes  Season:

(*one or few hooks*) Habitat: Target:

Purse seine Only  Mostly  Sometimes  Season:

(*or surround* *nets*) Habitat: Target:

Beach seine Only  Mostly  Sometimes  Season:

Habitat: Target:

Trawl nets Only  Mostly  Sometimes  Season:

(*or other towed net*) Habitat: Target:

Traps Only  Mostly  Sometimes  Season:

Habitat: Target:

Other (describe):

Only  Mostly  Sometimes  Season:

Habitat: Target:

48. In what places do you normally fish?

(*Use prepared road maps, charts, Google maps* *and have interviewee point out areas*)

Do you use different gears in different areas? Yes  No  If yes, please describe:

(*Use prepared road maps, charts, Google maps, and have interviewee point out areas*)

49. Do people in your village / community target sharks? Yes  No  No, but they are occasionally landed

Please elaborate:

(*Note to interviewer: This can be* *area sensitive*)

**Sea turtle catch / bycatch**

(*Optional if time permits and interviewee is keen*)

50. Have you ever seen sea turtles? Yes  No  Do you have another name for them?

51. What species of turtles do you see? Green  Hawksbill  Olive Ridley  Loggerhead

Flatback  Leatherback  Don’t know

Do you know the difference between these turtle species? Yes  No  Don’t know

(*Note to Interviewer; Show ID chart or graphics*)

Please describe:

Do they have different names? (if yes) Please list: (*determine for each species*)

52. How long do you think a turtle lives? Don’t know

53. How do you see turtles? Seen while fishing  Seen while travelling to fishing areas

Coming ashore to lay eggs  Accidentally caught in nets  Hunted  Stranded on the beach

(*Note to interviewer: Refer to and complete attached table and mark all locations on maps*)

54. How frequently have you seen turtles? Never  Once in my life  Only a few times in my life

Frequently  Every year for the last five years

In the last year, only once  several times  every month  every week  every day

55. When do you see turtles? (*indicate months or seasons*):

56. When was the last time you saw one? (*if long time ago note the year*)

57. Do you know of any areas where turtles regularly occur? Yes  No

(*Note to interviewer:* Regular *means certain times of year when they are always found*. *Indicate on maps*)

58. Do these turtle areas change over time? Yes  No  Don’t Know

59. How many turtles do you think might live in these areas? <10  >10  >100  Don’t Know

60. Do you see mating turtles? Yes  No  When? Where (*use maps*)?

61. Do people from other villages / communities catch turtles? Yes  No  Don’t Know

(*if yes*) How many (people)? What village?

Is the catch accidental or on purpose? Accidental  On purpose  Both

62. Do people in your village / community catch turtles? Yes  No  Don’t Know

(*if yes*) How many (people)? For how long?

Is the catch accidental or on purpose? Accidental  On purpose  Both

63. Did you personally catch any turtles in the last year? Yes  No

(if yes) How many in the last year? 1-2  ≤10  >10  Specifics (*if available*):

Was this is a typical number to catch in a year? Yes  No

(if no) Was it higher or lower than usual? Higher  Lower

Was the catch accidental or was it something you were fishing for? Accidental  Hunted  Both

64. Did you catch any in the last five years? 0  1-2  ≤10  >10  Specifics (*if available*):

How many in your life? 0  1-2  ≤10  >10  Specifics (*if available*):

65. How did you catch them? Harpoon  Nets  Other  Please describe:

When did you catch them? (*what month(s)?*) Where? (*ask interviewee to* *show on maps*)

66. Compared to when you started fishing, are there more , less , or the same number of  turtles

captured in fishing gear? Don’t Know  (*Note: based on actual numbers, not perception*)

(if more or less) Why do you think this?

67. What do you (or would you) do with a sea turtle if you caught one on purpose?

Eat  Sell  As Bait  Other Use : *(Note: do not lead interviewee)*

68. What do you (or would you) do with a sea turtle if you caught one accidentally?

Discard (*dead*)  Release (*alive*)  Eat  Sell  As Bait  Other Use :

69. Have you ever found  or heard of  turtles stranded on the shore? Yes  No

Or have you ever found  or heard of  turtles dead in our waters? Yes  No  (*explain stranded*)

Or have you ever found  or heard of  turtles with cut marks on their backs? Yes  No  (*explain*)

(if yes) Where, when and how many? (*ask interviewee to* *show on maps*)?

What happened to the animal(s)?

70. What would you do or did you do if you found a stranded turtle?

71. Compared to when you started fishing, do you think there are more turtles , less , or the same

number of turtles ? I don’t know

(if more or less) Why do you think this?

(*Note to* *interviewer: Try to determine what other impacts may be driving the trend*)

72. Do you think there will always be turtles in our waters? Yes  No  Don’t Know

(if yes or no) Why?

73. Do you think having turtles around is important? Yes  No  Why?

74. It is illegal to intentionally kill a turtle? Yes  No  Don’t know

What about by accident (maybe caught in a net unintentionally)? Yes  No  Don’t know

75. Are there any local customs, beliefs, legends or rituals or stories related to turtles?

Yes  No  (if yes) Please describe:

Where / from whom did you hear this?

**dolphin catch / bycatch**

(*Optional if time permits and interviewee is keen*)

76. Have you ever seen dolphins? Yes  No  Do you have another name(s) for them?

(*list by species*)

77. What species of dolphins do you see (*describe*)?

Any other cetaceans (*describe*)? Don’t know

78. How long do you think dolphins live? Don’t know

79. How do you get to see dolphins? While fishing  While travelling to fishing areas

Accidentally caught in nets  Hunted  Stranded on the beach

Are these areas different by species? Yes  No  Don’t Know

(if yes) Please explain:

(*Note to interviewer: mark locations on maps by species*)

80. How frequently have you seen dolphins? Never  Once in my life

Only a few times in my life  Frequently  Every year for the last five years

In the last year: Only once  Several times  Every month  Every week  Every day

81. When do you see dolphins? (*indicate months or seasons*):

82. When was the last time you saw one? (*if long time ago note the year*)

83. Do you know of any areas where dolphins regularly occur? Yes  No

Where are these dolphin areas?:

(*Note to interviewer:* Regular *means certain times of year when they are always found*. *Indicate on maps*)

84. Do these dolphin areas change over time? Yes  No  Don’t Know

85. How many dolphins do you think might live in these areas? <10  >10  >100  Don’t Know

86. Do people from other villages / communities catch dolphins / other marine mammals?

Yes  No  Don’t Know

(*if yes*) How many (people)? What village?

Is the catch accidental or on purpose? Accidental  On purpose  Both

87. Have people in your village / community ever caught dolphins / other marine mammals?

Yes  No  Don’t Know

(*if yes*) How many (people)? For how long?

Is the catch accidental or on purpose? Accidental  On purpose  Both

88. Did you personally catch any dolphins in the last year? Yes  No

(if yes) How many in the last year? 1-2  ≤10  >10  Specifics (*if available*):

Was this is a typical number to catch in a year? Yes  No

(if no) Was it higher or lower than usual? Higher  Lower

Was the catch accidental or was it something you were fishing for? Accidental  Hunted  Both

89. Did you catch any in the last five years? Yes  No

0  1-2  ≤10  >10  Specifics (*if available*):

How many in your life? 0  1-2  ≤10  >10  Specifics (*if available*):

90. How did you catch them? Harpoon  Nets  Other  Please describe:

When did you catch them? (*what month(s)?*) Where? (*ask interviewee to* *show on maps*)

91. Compared to when you started fishing, are there more , less , or the same number of  dolphins

captured in fishing gear? Don’t Know  (*Note: based on actual numbers, not perception*)

(if more or less) Why do you think this?

92. What do you (or would you) do with a dolphin if you caught one on purpose?

Eat  Sell  As Bait  Other Use : *(Note: do not lead interviewee)*

93. What do you (or would you) do with a dolphin if you caught one accidentally?

Discard (*dead*)  Release (*alive*)  Eat  Sell  As Bait  Other Use :

94. Have you ever found  or heard of  dolphins stranded on the shore? Yes  No

(if yes) Where, when and how many? (*ask interviewee to* *show on maps*)?

Have you ever found  or heard of  dolphins dead in our waters? Yes  No  (*explain stranded*)

Or have you ever found  or heard of  dolphins with cut marks on their backs? Yes  No  (*explain*)

(if yes) Where, when and how many? (*ask interviewee to* *show on maps*)?

What happened to the animal(s)?

95. What would you do or did you do if you found a stranded dolphin?

96. Compared to when you started fishing, do you think there are more dolphins , less ,

or the same number of dolphins ? I don’t know

(if more or less) Why do you think this?

(*Note to* *interviewer: Try to determine what other impacts may be driving the trend*)

97. Do you think there will always be dolphins in our waters? Yes  No  Don’t Know

(if yes or no) Why?

98. Do you think having dolphins around is important? Yes  No  Why?

99. It is illegal to intentionally kill a dolphin? Yes  No  Don’t know

What about by accident (maybe caught in a net unintentionally)? Yes  No  Don’t know

100. Are there any local customs, beliefs, legends or rituals or stories related to dolphins?

Yes  No  (if yes) Please describe:

Where / from whom did you hear this?

**confidential interviewer comments**

101. How open and honest did the fisher seem about answering bycatch questions?

Very open/honest  Somewhat open/honest  Not honest

102. How interested and engaged did the fisher seem with interview?

Very interested  Moderately interested  Bothered/ Not interested

103. How certain did the fisher seem about answers to numerical questions?

Very sure  Reasonable sure  Unsure

104. How comfortable were you about the respondents' ability to discriminate between the species

Very comfortable  Reasonable  Not comfortable

105. Why do you think this?

106. Please indicate why (if any) questions were not asked

| Survey ID Number | Sighting Record # | #  Individuals  seen | Habitat | Size  S,L | Mother - Calf Pair  Y / N | Day / Night | Year | Month | Dead / Alive | Cause | Condition | Accidental / Direct | Reported Y / N | Notes |
| --- | --- | --- | --- | --- | --- | --- | --- | --- | --- | --- | --- | --- | --- | --- |
|  |  |  |  |  |  |  |  |  |  |  |  |  |  |  |
|  |  |  |  |  |  |  |  |  |  |  |  |  |  |  |
|  |  |  |  |  |  |  |  |  |  |  |  |  |  |  |
|  |  |  |  |  |  |  |  |  |  |  |  |  |  |  |
|  |  |  |  |  |  |  |  |  |  |  |  |  |  |  |
|  |  |  |  |  |  |  |  |  |  |  |  |  |  |  |
|  |  |  |  |  |  |  |  |  |  |  |  |  |  |  |
|  |  |  |  |  |  |  |  |  |  |  |  |  |  |  |
|  |  |  |  |  |  |  |  |  |  |  |  |  |  |  |
|  |  |  |  |  |  |  |  |  |  |  |  |  |  |  |
|  |  |  |  |  |  |  |  |  |  |  |  |  |  |  |
|  |  |  |  |  |  |  |  |  |  |  |  |  |  |  |
|  |  |  |  |  |  |  |  |  |  |  |  |  |  |  |
|  |  |  |  |  |  |  |  |  |  |  |  |  |  |  |

*Habitat Codes: (D) Deep Water; (C) Coral; (S) Seagrass; (F) Fine Sediments; (M) Mangroves; (R) Rocks; (E) Estuaries; (U) Unknown*

*Cause: (G) Gill net, (O) Other Fishing Gear (specify in notes), (B) Boat Strike, (H) Hunting (D) Don’t Know*

*Condition: (F) Fresh, (D) Decomposed*
