# Supplementary material for: A low-cost solution for documenting distribution and abundance of endangered marine fauna and impacts from fisheries
Source: PLoS One. 2017 Dec 28;12(12):e0190021. doi: 10.1371/journal.pone.0190021 (PMC5746211; doi:10.1371/journal.pone.0190021)

United Nations Environment Programme

Convention on the Conservation of
Migratory Species of Wild Animals

UNEP/CMS OFFICE – ABU DHABI
United Arab Emirates

Dugong Questionnaire Survey

Project Manual

CMS-UNEP Abu Dhabi Office

September 2012

Acknowledgements

This document was produced following numerous discussions amongst project participants from projects in Southeast Asia, the Pacific Islands, the Southwest and Northwest Indian Ocean regions, the first South Asia regional meeting, and from an expert panel at workshop in Singapore to develop the Dugong Questionnaire, and by invited experts around the world subsequent to the development of the dugong questionnaire. We are particularly indebted to the input and counsel on project design by Helene Marsh, Mariana Fuentes, Patricia Davis, Ellen Hines, John Ben, Loiusa Ponnampalam, Grant Murray, Jeff Moore, Rebecca Lewinson, Konkiat Kittiwattanawong, Kanjana Adulyanukosol, Alana Grech, Jillian Grayson, Alana Grech, Rachel Groom and Lui Bell.

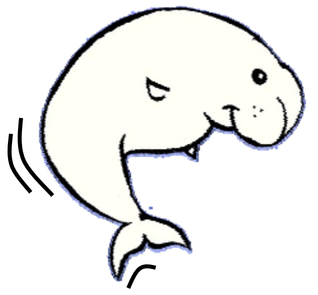

This document may be reproduced free of charge to assist with the implementation of the CMS-UNEP Dugong Survey, and should be cited as:

Pilcher, N.J & D. Kwan, 2012. Dugong Questionnaire Survey Project Manual. CMS-UNEP Abu Dhabi Office. United Arab Emirates. September 2012. 44 pp.

For further details or information please contact:

Dr. Nicolas Pilcher – Technical Advisor Email: npilcher@mrf-asia.org
Dr. Donna Kwan – Dugong Programme Officer Email: dkwan@cms.int

UNEP

UNEP/CMS Office – Abu Dhabi
c/o Environment Agency – Abu Dhabi
Al Mamoura Building A, Al Muroor Road (Street No. 4), P.O. Box 45553, Abu Dhabi, United Arab Emirates
Tel: +971 2 6934 437/523; Fax: +971 2 4997252; Email: CmsOffice.ae@cms.int; Website: www.cms.int

TABLE OF CONTENTS

1.0 Introduction 3
2.0 Rationale 4
3.0 CMS-UNEP Dugong Questionnaire Survey 4
4.0 Manual Layout and Purpose 5
5.0 Interview Methods and Techniques 6
6.0 Data Integrity 11
7.0 Dugong Interview Survey Design 14
7.0 Dugong Interview Survey Design 14
7.1 Effort and Efficiency 14
7.2 Stratified Sampling 16
7.3 Random Sampling 18
8.0 Field Data Collection and Control 21
8.1 Serial Numbers on Surveys 21
8.2 Entering Data on the Table 22
8.3 Use of Graphics and Field Guides 23
8.4 Linking Graphics to Table Data to Survey Numbers 25
8.4 Linking Graphics to Table Data to Survey Numbers 26
8.5 Training Requirements 28
8.6 Miscellaneous Questionnaire Details 30
9.0 Uploading Graphics and Spatial Data 31
9.1 Creating Google Earth Layers 31
9.2 Adding Sighting / Stranding Records 32
9.3 Adding Fishing and Seagrass Areas 33
9.4 Saving our Google Earth Files in .kmz Format 34
9.4.1 Saving Sightings data in .kmz Format 35
9.4.2 Saving Individual Fishing and Seagrass Areas as .kmz Files 36
10.0 Literature Cited and Additional References 38
Annex I – Sample Field Guides 39
Annex II – Sample Seagrass Field Guide 41
Annex III – Sample small- and large-scale maps 43

1.0 Introduction

The Dugong (*Dugong dugon*; Family Dugongidae), is a seagrass-dependant marine mammal found in tropical and subtropical coastal waters. It is the sole member of the genus Dugong, which is the only extant species of the Family Dugongidae (Husar 1978; UNEP 2002). The Dugongidae share the Order Sirenia with three extant species of manatee.

Dugongs, also known in many areas as sea cows, are found in the warm tropical and subtropical coastal and inland waters of the Indo-Pacific. Their distribution is broadly coincident with the tropical Indo–Pacific distribution of its dietary components, seagrasses in the families Potamogetonaceae and Hydrocharitaceae (Husar 1978).

Although dugongs occur over 40 countries, most of these are developing economies with limited capacity to contain impacts on dugongs within sustainable levels (Marsh 1997), and population declines and local extinction have been reported from a number areas within their range. The International Union for the Conservation of Nature (IUCN) rates their extinction risk as Vulnerable on a global scale. This risk is based on an inferred or suspected reduction of at least 30-50% over the last three generations (90 years; (Lawler et al. 2002). This classification describes a taxon that faces a moderate risk of extinction in the wild within 50 years (Marsh 2008). A recent report on status of dugongs throughout their range with the assistance of more than 100 experts indicated that dugong populations are declining or extinct in at least one third of its range, of unknown status in about half of its range and possibly stable in the remainder – mainly the remote coasts of the Northern Territory and Western Australia (UNEP 2002; Marsh 2008).

Dugongs are vulnerable to two broad classes of impacts: those that kill animals directly, for example netting, traditional hunting or large-scale losses of seagrass, and those that decrease the calving rate by reducing feeding opportunities, for example smaller-scale habitat loss or boat traffic (Marsh 1997). Among these, entanglement is the predominant threat to dugongs as they are by-caught in many kinds of fishing gear, in both commercial and artisanal fisheries. The threat is considered major, but the magnitude of the impact is largely unquantified in many countries (Perrin et al. 2002). Little reliable information documents these impacts (UNEP 2002; Poonian et al. 2009), particularly because much of the dugong's range is in developing countries which often lack the necessary resources to conduct resource- and finance-demanding surveys (Aragones et al. 1997). Therefore, there is still a critical need to survey and assess marine mammal populations, and quantify artisanal fishing effort and by-catch of marine mammals in these data deficient areas (Aragones et al. 1997; Poonian et al. 2009).

2.0 Rationale

A number of sophisticated survey techniques have been developed for marine mammals. These include conducting line transect surveys from ships or boats and airplanes. However, these methods often have limited application in developing countries, because they require expensive and sophisticated equipment, trained support personnel, or cost too much (Aragones et al. 1997). But there are several lower-cost survey techniques for marine mammals that are applicable in developing countries, such as interview surveys, land/shore based monitoring, and carcass analysis (Aragones et al. 1997). These more economical surveys, such as interviews, are ideal for initial surveys in areas where there is little or no information (Marsh and Lefebvre 1994; Aragones et al. 1997). Importantly, there is a need to link population demographic data with spatial information on dugong, dugong habitat and fishery distribution.

3.0 CMS-UNEP Dugong Questionnaire Survey

Under the auspices of the CMS-UNEP Dugong Memorandum of Understanding, a dugong-oriented questionnaire-based survey (which can also collect data on marine turtles and cetaceans) was developed by an expert panel using the outcomes of various past interview survey initiatives. The survey was based in large part on the revised protocols developed by the Project GLoBAL Rapid Bycatch Assessment (http://bycatch.env.duke.edu/) but also drew on protocols developed at the Phuket Marine Biological Center (Thailand), at San Francisco State University (USA) and at James Cook University (Australia). The multi-disciplinary background of the panel ensured that the survey design would be widely applicable across regions and issues, scientifically thorough and sound, and culture-sensitive. The survey protocols were then reviewed by a number of social science and bycatch assessment experts to determine language appropriateness and scientific rigor.

The results of the surveys can assist in determining the distribution and abundance of dugong populations, help identify and map areas of important dugong habitat such as sea grass beds, and assess the risk of, and develop measures to mitigate degradation of dugong populations and their habitats. The standardised survey protocol can also be of great benefit for comparisons within and across regions. The survey protocol can be implemented rapidly and at low cost, has the required scientific rigor to be quantifiable, links spatial and demographic data, is easy to report and flexible in its approach.

This manual addresses a suite of factors relevant to the implementation of the Dugong questionnaire survey.

4.0 Manual Layout and Purpose

The references to implementation methods, sampling design and interview techniques contained herein are not intended to be all-inclusive as there are a range of extremely valuable and thorough documents which address project design (e.g. Fowler 2009). This manual is intended only as an abbreviated summary of key aspects of project implementation, to guide implementing individuals / agencies in the inception and conduct of their individual projects.

This manual is an evolving document, and experiences in the implementation of this project in various range-state subregions have already contributed to various revisions. It is important to note that suggestions made for the implementation of this project in this manual should be taken in a cultural and geographical context: what may be applicable in one scenario may be irrelevant in another. It is the responsibility of the implementing agency / individual to optimise the spatial coverage and thoroughness of the survey (number of interviews) so that the results can be considered statistically representative of the areas / ports / communities canvassed as part of this project.

The project implementation aspects of this manual are divided into several sections. The section on *Interview Methods and Techniques* addresses some common and critical aspects of questionnaire-based survey implementation and highlights common pitfalls. The section on *Data Integrity* is concerned primarily with confidence around interview results, and interviewer and interviewee integrity and reliability. The section on *Survey Design* is intended as a preliminary guide to setting up a project with the requisite spatial coverage and which will provide sufficiently-robust data sets for subsequent national and regional analysis. The final section on *Field Data Collection and Control* is intended to provide a simple guide to the use of the Survey and its accompanying Table, how to use (appropriate and absolutely essential) graphics and guides, and how to relate all data both geographically and back to the original questionnaire. These sections provide guidance only and are no substitute for thorough project planning taking into account all social, cultural and geographic variables.

This Project Manual probably looks a little like the Instruction Manuals that come with a brand new digital camera. There are a bunch of headings you think you know all about and want to skip, and you really want to jump straight to the field implementation section (Section 8.0). But then you'd be missing the most valuable lesson imparted by all professional photographers: "RTM" or "Read the Manual". There are many insights into sampling, why things should be done one way or another, and why your results could suffer if things are not done and planned correctly. We urge you to take some time and go through it all.

5.0 Interview Methods and Techniques

Good interview techniques require practice so project staff should not expect to master them immediately. All project staff should have conducted a minimum of ten trial interviews before heading out into the field. Project staff will be expected to have a basic knowledge of the subject – dugongs, turtles, cetaceans, bycatch, and should not conduct an interview with a fisher / relevant person without this background information. If they show their ignorance, they will lose credibility and risk losing the confidence of the Interviewee (person being interviewed). At the very least, the Interviewee is less likely to open up to them and provide truthful, reliable answers. It is the responsibility of the Project Manager(s) to provide the interview staff with the basic background knowledge to effectively conduct interviews in the field, and the opportunities to conduct trial interviews with other staff or volunteers. Interviewing is very closely associated with psychology. The better the staff understand how people think, the better they will be able to extract their thoughts from an interview.

Interviewing respondents is a challenging task. One of the most challenging aspects of this work is gaining the trust of the respondent in a very short period of time. Professional social scientists might spend months or years integrating with local communities, a time frame that is not available to us in this work. We need to understand the challenges and limitations of interviews with people we have only just met.

Some respondents are hesitant because they fear reprisals through their responses – for instance, if a fisher provides an indication that stocks are collapsing and in danger, government may decide to impose fishing restrictions which may not be in the fisher's better interests. Similarly, if a fisher has caught a dugong and he / she knows it is illegal, they may fear fines or prosecution from enforcement authorities. It is the responsibility of the Interviewer (the person asking the questions) to set the Interviewee at ease, and allay any fears they may have from their participation. This is not an easy task, and less so given the relatively rapid nature of this sort of survey. This is the reason all interviews start with an **Introductory Statement**. It is important that all Interviewers read this statement carefully so that the Interviewee understands why they are being asked questions, and what the consequences of their involvement might be. It is also ethically appropriate to let the subject know what their responses will be used for.

The Dugong Questionnaire Survey, by design, is intended as a relatively rapid process of accumulating data which addresses spatial distribution (where things are and where fishery pressure is located), and population status (how many are there, and how many were there in the past, and what sort of protection measures are in place). The Survey is also intended to be

implemented at low cost. That is, it is not intended that only qualified PhD holders in social sciences undertake the survey – rather, it is intended that graduate students, research assistants and NGO staff conduct the interviews with some basic training and background information. The following tips are very general and apply differently to different situations. The Interviewer should use his / her judgment to decide when and how to use them:

Dressing Appropriately – Appearance will influence the way Interviewees respond to you. If the Interviewer appears too official it is possible respondents will not feel comfortable. Similarly however, a very informal dress code might be equally inappropriate. Lastly, it is important for women who are conducting interviews to fit in with the customs and norms of the community in which they are conducting interviews, and for all interviewers to respect the customs and cultures of the people they are interviewing.

Trying to be Unique – It helps if the session is not just another interview going over the same questions the subject has answered many times before.

Being Honest – Sometimes it is tempting to lie or omit important information when securing an interview. This is more than just unethical; it will undermine the Survey process in the long run.

Staying Neutral – It is important to try not to demonstrate any bias. The Interviewer should not appear to be persuaded by the subject's opinions and not judge or directly criticise the subject.

Interrupting – This can upset the Interviewee's train of thought.

Over-Directing – The Interviewer should try not to give the subject too many instructions or be too specific about what they want them to say. In most cases it is better to let them speak freely. Over-directing also often results in leading questions (see this Section, a bit further down).

Showing Understanding – The Survey often covers sensitive topics. The Interviewer should show some understanding for the subject without getting overly-linked to one side of an argument, and ask for permission before asking difficult questions, e.g. "Is it okay to talk about...?"

Missed Questions – If the Interviewer missed a question from the interview, he / she might be able to revisit the subject and get the answer. But generally we only get one chance at this — ask too many times and we will probably be out of luck.

Because of the rapid nature of the Survey, and the fact that it will often be implemented by project staff with limited training, it is important that the following points are taken into consideration:

Respect the Interviewee – It is important that the Interviewee (the person being interviewed) feels that he / she is respected and that their information is valuable. There are times when the person who asks the questions may look at the surroundings and feel that a fisher is poor and may have limited knowledge and understanding, but this must be avoided at all costs. What a fisher understands about fishing and the ocean are generally far in excess of what any interviewer will know, and this knowledge, while different, needs to be respected. In most instances, with respect comes honesty. If an Interviewee is treated with respect, there is a much greater chance the responses will be truthful.

Allay any Fears – As noted above, a person may be intimidated by the process of being interviewed, and fearful of reprisals. As stated in the **Introductory Statement**, "…Individual answers will be collated and reported on as a group to provide a general idea of current status, and we will absolutely not share your individual answers to anyone outside of the research team…", so it is not the intention of the survey to use individual answers, but rather to synthesis these and determine overall trends and activities. **Individual** survey results will **never** be shared with people from outside the survey team, including the authorities, and all information from individuals will be treated with confidentiality. It is important that this message be transmitted to the Interviewee.

Explain Exactly How the Interview Results will be Used – It is important to explain that an individual data set does not really tell us much about what the big picture is. What one fisher sees may be only a fraction of what a large group of fishers, from a variety of ports, see. Therefore it is important to explain that the data will be summarised and that general distributions of fishing effort, threats and animal distribution will be amalgamated across all interviews. The results will then be used to look at areas where both threats and dugong / turtle / cetacean populations overlap (these are also called data later overlays) and the project will use the results to develop conservation initiatives which are beneficial to both marine fauna and fishers alike.

Explain What Impact the Results Might Have on the Interviewee – Not only is this a project requirement, it is ethically the right thing to do. It would be unfair to take a respondent's data and suggest no harm would come of the process, if the ulterior motive was to initiate conservation action around the dugongs / turtles / cetaceans, which might result in restrictions / negative impacts to the community. While this honest and truthful approach might dissuade some people form participating, this is a risk the project has to take,

and record (see Section 8.6). We recognize that the overall aim of the project is to conserve dugongs, other mammals and sea turtles. This is achieved normally through fishery restrictions, gear type modifications, time-area closures, and the like. But these can be detrimental to fishing communities (for example through costs of new gear, or additional fuel to travel to unrestricted areas), and we (the project implementing team) must be sensitive to this fact. While it is unlikely the results of this process alone will result in drastic negative impacts to communities, it is important that they realise what the ultimate objectives are. But similarly, it is important that they understand that our goals are to develop solutions which are a win-win situation – for marine wildlife and also for communities.

Singular vs. Group Interviews – Interviews can be conducted in a group setting or with individual respondents. Sometimes people feel more relaxed and speak more freely when surrounded by their colleagues, but others feel it is easier to be frank when speaking in private. Private discussions work best for this type of interview, but responses from one individual can still be distilled from a group discussion. It is important when speaking to a group of people that the responses of one individual alone are captured, and not that of a number of respondents on a single survey form. As an example, one could return to the key person after each question has been deliberated by saying "And Chong (or Pete, or Abdu, etc.), what to you think about this?"

The Use of Voice Recorders – Recordings have been found useful in revisiting responses by individuals to make sure the data sheets are correctly completed. It is acceptable to record the interview with an electronic voice recorder, but always with permission. Some Interviewees may be disturbed by the presence of the recorder, and think that it could be evidence which could be used against them. Others may be shy. The Interviewer must always ask if the Interviewee is agreeable to having the session recorded, and sensitive to the fact many people will refuse. Some people find that covering the recorder with a piece of paper or cloth eventually allows the respondent to subconsciously ignore the fact it is there.

Listening – A common mistake is to be thinking about the next question while the subject is answering the previous one, to the point that the interviewer misses some important information. This can lead to all sorts of embarrassing outcomes.

Leading Questions – A leading question is a question which subtly prompts the respondent to answer in a particular way. Leading questions are generally undesirable as they result in false or slanted information. Questionnaire interviews are designed to capture the responses by the Interviewee, not a proposed response by the person asking the questions.

When an answer is provided as part of a question, this is called leading the Interviewee. For example, if you were to ask “When did you last see a dugong, last week?” you are already suggesting an answer (last week) as part of the question. It is a known fact that respondents will often choose the suggested answer rather than provide one of their own when undecided. If you use Leading Questions, you will end up with biased responses to the question, particularly if you do it in each case. A question worded as “What would you do if you caught a dugong? Release it?” will more often than not get a “Release” response rather than “I would eat it” response. It is important to ask all questions neutrally and seek the personal response from the Interviewee. If this response is not listed as an option on the Survey form, please write it down as a note.

Open-Ended Questions - An open-ended question is designed to encourage a full, meaningful answer using the subject's own knowledge and / or feelings. It is the opposite of a closed-ended question, which encourages a short or single-word answer. Open-ended questions also tend to be more objective and less leading than closed-ended questions. Many of the Survey form questions are closed, but there are several open-ended questions which require greater attention than closed questions. It is important (in the interests of respect and inclusion) to allow the respondent to answer these fully, but it is also important (in the interests of time) to keep the discussion on track and to be able to move on and complete the questionnaire.

Use the Form – Many people believe they can remember the questions and fill in the form later, and feel more comfortable not having the paper in front of them. But it simply does not work amongst untrained personnel. Using the Standardised Survey Form demonstrates a degree of professionalism, rather than simply ‘having a chat’ with the Interviewee, and it is likely the responses will reflect the situation more accurately by addressing points in sequential order rather than through a friendly meandering conversation. It is important that the Interviewer hold the Survey form and go through the questions one by one, ticking and answering where appropriate, so that no questions are omitted and all answers are recorded accurately.

Use Maps – Possibly the most important part of the survey is how it is linked to spatial data (the ‘where’ in every question). Any time there is a question which relates to a place, it must be marked on the map (see also Section 8.3). No data on a map means only a small fraction of the results have been achieved. You must have the maps right in front of you the whole time you interview someone, and mark sightings, strandings, fishing areas, seagrass areas and any other important spatial data on the map as the interview progresses. It is often best to ask the fisher to make the map him / herself.

How to End the Interview– When the interview is finished, the Interviewer should put the Survey form away (and recorder if used) and have an informal chat. As well as being polite and leaving a good impression, one might be surprised at what additional information flows when the subject thinks it is all over and is more relaxed.

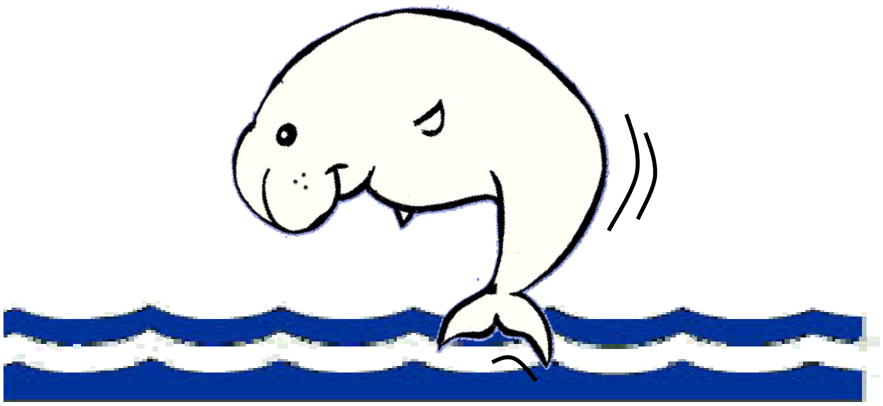

6.0 Data Integrity

In this context Data Integrity relates to how robust and accurate the data collected by the project staff are, and how they need to be handled to ensure we get the most reliable indication of current population status, trends, distribution and impacts. It is important to keep in mind that the project is designed to empower a team of staff / students / volunteers with basic training to go out and collect data which has to be converted into statistically-valid and geo-referenced information. This is no easy task, and several points need keeping in mind as the project is designed and implemented:

Reliability of the Interviewer – When the Interviewer is sent out into the field, he or she is alone and has much of the control over what data is collected, and how data sheets are completed. It would be easy to invent the data and spend time in a café drinking coffee or tea, and so it is paramount that the Project Manager(s) explain with care just how important the process is, and what value the data have. Interviewees should be given an understanding of how the Survey will assist with the conservation of marine fauna, and made to understand just what a valuable role they play in the process. The more the project staff feel they are involved and valued, the more likely it is that they will collect data in a reliable and professional manner.

Representative Value of an Interview – One interview will not tell us much, but a number of interviews in one location will provide us with a glimpse of the actual situation. Interviewers (project staff) will have to make on-the-spot

decisions as to the requisite number of interviews (see also Section 7.2) if conditions change from what is expected.

Thoroughness – It is the responsibility of the Interviewer to ask all questions on the Survey (except those optional ones on turtles and cetaceans – and even these should be asked when possible) and indicate where questions have not been asked (tick to the left of the question) and provide suitable explanations for this in Question 107. It is also the responsibility of the Interviewer to ensure that a thorough and representative sample of fishers from each location is approached. Using a stratified sampling approach (see Section 7.2), the interview team needs to target a proportion of the larger ports, a proportion of the smaller ports, a proportion of the irregular landing sites, and a proportion of any other level of fishing port or site. If the team could target 10% of fishers in the areas it went to, they would provide really good coverage but this is likely not practical all the time. For instance, 10% of a village with ten fishers is only one person and the results would not be representative. A sample of 10% of 2000 fishers might be overkill as the team will reach a point where they learn nothing new even if they continue to push for the 10% target (see Section 7.1). It would be good to keep in mind the idea of "there is little or no added learning from surveys that all tell you the same thing", so that when the team is out in the field they can move on if everyone they interview starts to says the same thing. The overall objective is to provide an updated picture of the current state of the dugong population in each area upon which the decisions and prioritised action can be developed, not to know the slightly varied fishing locations of 200 or 300 fishers. Project management can provide initial guidance, but in the field the Interviewer has to assume a degree of responsibility towards project outcomes.

Independence in the Field vs. Supervision – When project staff are in the field they are unsupervised. It is critical that they have the integrity to collect data in a professional manner without the need for supervision, as often the supervisors can impact the response rate and behaviour of the Interviewee. For instance, if a senior Fisheries official is lurking in the background, fishers may be uncooperative or timid. Junior staff (and better yet students or NGO staff) typically are much more openly received / welcomed than top government officials, particularly given the rapid format of this interview approach. It is important that project managers convey a degree of trust in the field staff and empower them to act without supervision, and impress upon them the need for reliable data sets upon which to base conservation action.

Transfer of Data to Electronic Format – Electronic upload files developed by CMS-UNEP (http://www.cms.int/species/dugong/index.htm) for use with the Quastionnaire Survey are of great help, and their use is required if the data

are to be easily analysed and uploaded into spatial analysis (GIS) packages. However, it is important to ensure the data which is transferred to electronic format is the same as that on the data sheets. This can be checked by random sampling the data sheets against the actual data sheets by an independent person. For example, a staff member not involved in data upload could check every (e.g.) tenth sheet against the computer records for accuracy. If errors were detected, several additional sheets would need to be checked to find out if the errors were frequent. This process will ensure that what is collected in the field is what is analysed further down the road.

Compounded Errors – This project intends to use individual records to develop a community picture, and then take community pictures to develop a National picture, and then take National pictures to develop a Regional Picture. If an error is introduced at the bottom of the chain, it remains in the system all the way through. Imagine telling a government official that Area X is a critical habitat only to find out there are no dugongs there! It is important that errors are identified early and fixed, rather than have them paint an erroneous picture of conservation needs. Not only would this be a waste of conservation resources, but it would also be extremely embarrassing.

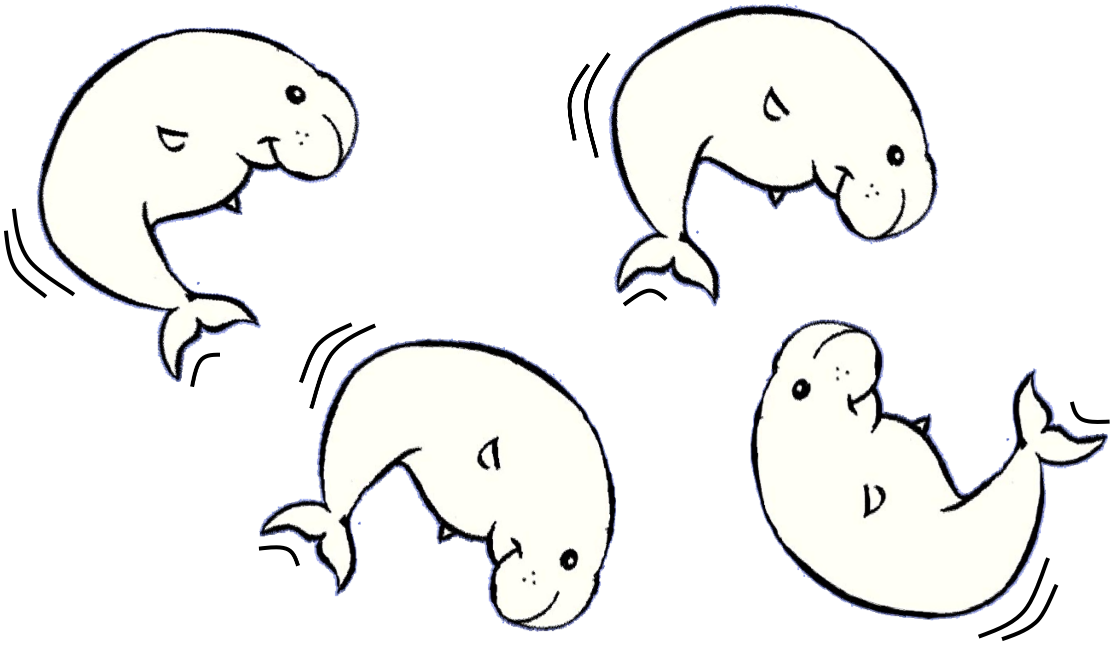

7.0 Dugong Interview Survey Design

The design of the Survey – how many interviews, where, for how long, etc. are key aspects of this survey process. Insufficient coverage will not yield statistically valid responses, and over-sampling is a waste of valuable resources. Where does one draw the line? This section introduces several concepts which will assist in developing a suitable framework for project implementation: *Effort and Efficiency* addresses how much survey effort is required; *Stratified Sampling* describes how sampling should be distributed, and *Random Selection* highlights the unbiased selection of sites across sampling layers.

7.1 Effort and Efficiency

No survey wants to invest more effort than is needed, but similarly we do not want to send people into the field and have them collect insufficient data. Imagine being in a situation where a few additional surveys could have made the difference between useful data sets and statistically-invalid ones. With limited resources it is up to the Project Manager(s) to design a sampling strategy which will best spread resources across sites. Lots of data does not necessarily mean substantially better results. Once we know where dugongs are distributed in a region, having five more people tell us the same thing will not add greatly to our knowledge. Undeniably it adds to the reliability of the data, which is a good thing, but with limited resources we might want to invest that effort in finding out about other areas. The following figure graphically shows how this happens:

As Effort increases initially (lower left), results go up substantially, reflected in the sharp rise in the curve. However, after a while the continued increase in effort does not lead to any major increase in Results (upper right). Imagine counting colours of cars passing in a two minute interval: Initially there would be reds and greens and blues and whites and grays of various tints. In the second interval there may be some additional colours, and in the third some more. But after a while you would have seen most of the colours, and the additional time intervals would only result in one new colour here and there. At this point you have reached the optimum sampling effort, and need to move on.

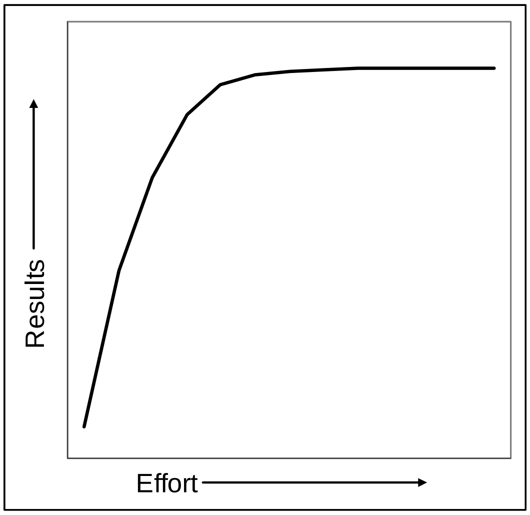

Optimally, each project should be seeking somewhere around a <u>10% coverage</u> of areas as a healthy target. However, we realize that at times this might be unrealistic, and that there will be a need to balance this with the size of the

community in question. We also are aware that fishing community sizes vary, and thus it is important to vary effort with community size.

For instance, if you are surveying a fishing community of two thousand fishers, two hundred surveys are likely to exceed that tipping point after which the extra data does not provide substantially new findings. In this case maybe only 3 to 5% coverage would be adequate. Fifty surveys is a lot of effort…

Conversely, in a small community of ten fishers, one interview will only tell you what one person knows, and this might not be representative of the overall community knowledge. In fact it is very likely to be biased one way or another. In this instance it would be advisable to speak to four or five of the key fishers. An absolute minimum of five to ten interviews at any one location are generally required to have a basic understanding of the situation.

It is imperative that effort is adjusted by site according to community size, but also that a degree of flexibility exists whereby the Interveiwer can decide enough data has been gathered and move on. This requires having a prior knowledge of the relative size of a fishing / coastal community, and a 'feel' for how much new information is being gained as the project progresses. This knowledge can be derived from past fishery studies, past interview surveys, or from any available relevant literature. In some cases it might be deduced through looking at road maps and finding out which communities are larger than others (a caution note: just because a village looks large on a road map does not mean there are many fishers). The initial phases of the project design must take into account a variety of information sources before settling on a sampling protocol. In general though, longer periods need to be scheduled for large communities and shorter visits to smaller communities.

Finally, at the design stage and during field implementation, both program managers and field staff need to use common sense! If the field team arrives at a site and finds that the fishing community has moved, or changed in size, then sampling effort needs to be redistributed. And importantly, any deviations from the original plan need to be carefully documented so that data analysts can interpret the results at a subsequent date. For example, if the team were supposed to conduct twenty interviews at one site but did not, and they did not report this clearly, the data analysis process would conclude that the work had not been done to a statistically significant level, and the data might be excluded from the overall analysis, which would be a tremendous waste of effort. If the process was well documented, and the size of the fishing community was known, the data would be of great use.

7.2 Stratified Sampling

In statistics, Stratified Sampling is a method of sampling from varying segments or categories of a population. When smaller components of a population (we can call them subpopulations) such as fishers from large fishing villagers compared to fishers from small fishing villages vary considerably, it is probably a good idea to sample each subpopulation independently – that is, sample some fishers from small villages and some fishers from large villages and treat them as different groupings. Stratification is the process of grouping members of the population into relatively similar subgroups before sampling. In our case we could categorise fishing communities by size, and by the amount of information we have on each of them.

We might want to group all the 'large' fishing communities (maybe all those with more than 100 boats), then we might group the medium-sized communities (possibly with 50 to 100 boats) and then smaller communities (maybe those with less than 50 boats). Of course, these are arbitrary selections, and each project will know better how to break down fishing communities or their relevant groupings. What is important is that each category should be mutually exclusive: that is, each element in the population (say, each fishing village) must be assigned to only one category. A fishing village with 50 boats can't be placed into the 100-boat category also. And the categories should also be collectively exhaustive: that is, no element can be excluded (all ports should fit into at least one of the categories). After this has been sorted out, random or systematic sampling is applied within each category by the data analysts. This often improves the representativeness of the sample by reducing sampling error, and it can produce a weighted (balanced where needed) mean that has less variability than the arithmetic mean of a simple random sample of the overall population. In real language what this means is that the averages of each *sub*-population are likely to be more representative than if we took one overall average of the *entire* population.

Stratification Based on Community Size – It is probably obvious that we can't quite compare the fishing pressure and potential impacts of all communities equally – larger communities are likely to exert greater pressures than smaller ones. Imagine that we find out that a community's fishing effort overlaps with important dugong habitats. This would be a concern if there were 50 boats, but probably not so much of a concern if there were only two boats. Or seen another way around, this might be a concern if there were 20 dugongs, but maybe not so much of a concern if there were 500 dugongs. Stratified Sampling allows us to determine the relative *weight*, or 'impact' of each category

You can use the following example to determine where you should be investing your effort to get the best out of your sampling effort. Just remember to change the numbers or categories accordingly!

If you had 10 large ports, 30 medium ports and 60 small ports, we would suggest an allocation of effort that reflected this breakdown, at a ratio of 10:30:60. For example, if you had resources to send a team out to sample 40 ports, how would you allocate effort? In this example you would take 10% of 40 to determine you needed to sample at 4 large ports (10% of 40), 30% of 40 to determine you needed to sample at 12 medium ports (30% of 40), and 60% of 40 to determine you needed to sample at 24 small ports (60% of 40). See the table below:

<table>
<tr><th># of Ports</th><th># of Ports to be Surveyed*</th><th>Calculating Proportions</th><th>Actual Calculations</th><th># of Ports to Sample</th></tr>
<tr><td>10 Large</td><td rowspan="3">40</td><td rowspan="3">(# of Ports / Total Ports) x Effort Available</td><td>= 10 / 100 * 40</td><td>4</td></tr>
<tr><td>30 Medium</td><td>= 30 / 100 * 40</td><td>12</td></tr>
<tr><td>60 Small</td><td>= 60 / 100 * 40</td><td>24</td></tr>
</table>

***Note: The number of ports to be surveyed should be determined based on the funding available / the number of interviews you think can be conducted with the resources you have.**

How you break down this allocation of effort is probably as important as the description of why you did this (and how) in the project reporting phase. Data analysts at a later date will need to know why you chose the particular four large ports, and the 12 medium ports, and on what you based your decisions. It is important that all such processes be carefully documented for clarity and transparency in data analysis, particularly if data are to be extrapolated later to represent greater geographical areas.

Stratification Based on Known Distribution – In a similar manner to small versus large communities, we should also stratify our sampling based on what we know and what we don't know. In many parts of the world today we have some idea of where dugongs are distributed or where they have been distributed in the past. There's not much point investing loads of effort in places where we know there are no dugongs. It is much more useful to invest effort in places where we know dugongs exist.

But at the same time, it is always a good idea to make sure that what we think we know is actually the current situation. What if dugong populations have moved since the last surveys? What if they were in such small numbers as to be undetectable in the past but now they have rebounded and people are seeing them? For this reason, we need to invest energy in both the places where we know (or have a reasonable reason to believe) dugongs are present,

and also in areas where we don't expect to find much but hope we will be pleasantly surprised. In other words, we need to confirm what we think we know, and find out what we don't know.

Even if the survey results return a 'No Dugong' result, or a 0 for the effort you invest, you have still made a valuable contribution to the survey. Knowing what is *not* there is just as important as knowing what *is* there. In science, a 0 is as valuable as a 1 or any other number. And every now and then you will find something completely unexpected….

As a general guide, we suggest you invest roughly 80-90% of your effort in surveying known areas and roughly 10-20% in surveying unknown areas which might reveal new information. This is again a general guide and not any sort of regulation, and we hope the project management in each location will be in a better position to make a final decision.

7.3 Random Sampling

Do you think it is at all surprising that there are always a higher proportion of boys at an all-boys school than at a mixed school? In fact, they are always boys! This is an example of biased sampling. Now, if you had sampled child height, or shirt colour, or arm length, these would not have been biased. There is a good chance there will be many boys of different heights or with different shirt colours and arm lengths. But every time we specifically choose some thing in a specific place or of a particular type or with a particular characteristic we are introducing what is called *bias*.

Bias occurs when sampling results are skewed in one direction or another because of a particular influence, and is something we want to avoid if we are to conduct truly random (and thus statistically-valid) sampling. There is no way we can interview everyone, so we need to choose a representative sample of people to interview. We need to choose a representative sample of ports to visit. This word, *representative*, is crucial to the outcomes of this project. If, for instance, we only chose to interview young members of a community we would never get any data on historic (past) trends. This is a *biased* survey. It is not *representative* of all people in the community. If we only interviewed elderly people in the village in the middle of the day, when most fishers were at sea, we would get biased results also. Neither would they be representative – these people may not be active fishers and may not have the best current information. Project managers need to select a cross-section of the fishing villages / community / fishers themselves to gather results which represent the widest range of possible responses.

Eliminating bias from our surveys is crucial so that the information we collect is not skewed (leaning) in one direction or another. Just as we don't want to collect information only from young, or old, or black-haired, or overtly-friendly fishers, we do want to make sure we get a little bit of everything, and a bit more from those who truly count rather over those who may not know as much (oops, there comes that Stratified Sampling again!). That is, we need a *Random* sample of people who are relevant to the project objectives. Or said in another way, we need a random sample of fishers or ports or fishing methods once we have decided within each grouping of our stratified approach. We *do* want to interview active fishermen and relevant workers who know about the marine environment. That means that we will need to concentrate on those people who spend most of their time at sea, and you could argue this is a bias as of in itself.

Random Sampling is not Unorganised Sampling – The survey design needs a random approach to selecting interviewees, to eliminate bias and to ensure the results are representative of the population being sampled. But random sampling is quite hard to get right. How does one conduct a random sample? How do you select which fisher to interview and which one not? What we don't want is for the Interviewer to just go out and ask anybody – this is unorganised sampling (aimless, unsystematic, very hit and miss). An unorganised survey might end up interviewing the bus driver, or a ship engine room mechanic, or a farmer. We want the Interviewer to approach relevant people, but in a random manner. In random sampling, each fisher or Interviewee in a population has an equal chance of being chosen at each selection. A sample is random if the method for obtaining the sample meets the criterion of randomness (each Interviewee having an equal chance at each draw). So how could we ensure this random selection in the field? Well, one option might be for you to identify how many fishers there are in a village and decide on interviewing every tenth one you meet. Another might be to interview people from every fifth boat at a fishing pier. Yet another might be able to get a list of all fishers by name, assign them a number (1, 2, 3 etc.…) and then to select the number of people you plan on interviewing by using a program (such as MS Excel) to pick that amount of random numbers (in Excel, you can enter =RANDBETWEEN(1,N) where N is the total number of names, to get a random integer number. The number returned by Excel is randomly selected, and can correspond to a person on your list.

To eliminate the bias and get a representative random sample, we need to balance these interviews then with a few other members of the community – women, older retired people, youngsters, etc. – to get an overall picture of current status. So how do we do this?

Eliminating Bias at the Site level – Some of this has already been dealt with the Section 7.2 on Stratified Sampling, but what is important to note is that the bias in selecting *sites* more often than not occurs in the project design phase, rather than in the implementation phase. When the Project Manager(s) sit down to decide which ports will get sampled, this is when bias can be introduced. For instance, imagine selecting only those sites which have good road access. Or only those sites with a hotel for survey staff to spend the night. Either of these two criteria suggest somewhere large and affluent, possibly somewhere where there is a Fishery Department office, where responses by Interviewees may be compromised. At the same time, there may not be that many of these types of sites, and therefore not all types of fishing communities will get sampled, and indeed only a small proportion of all fishing communities. It is important at the design phase to sample a good and representative cross-section of the entire coastal community.

Eliminating Bias at the Fisher level – Here again there are numerous ways of collecting biased information which can often be solved easily. The key difference is that the selection of fishers in this case is up to the Field Staff and not so much Project Manager(s). Because of this, it is imperative that training sessions deal with the issue of biased / random sampling so that the Field Staff do not return with irrelevant data sets. Imagine that the Field Staff arrive at a fishing community at midday, and plan on spending only two hours interviewing fishers. It is unlikely they will meet the fishers who are out at sea, and thus their sample will not be random. Imagine if they only select people with clean boats. Agreed, data sheets will not be as smelly, but maybe those fishers with clean boats are not really as knowledgeable as those out day to day on their (fish-smelling) boats. Common sense rules here, insofar as Field Staff need to be aware of the need for sampling a representative cross-section of the coastal community.

A Critical Need for Extrapolating Data – The concept of random sampling (at a stratified level) is not necessarily novel or rocket science. What it is, however, is a crucial need for this project to be able to extrapolate data across wider geographical ranges at a National level, and incorporation of these into Regional level analyses. If a coastal area did have the 10:30:60 Large:Medium:Small fishing communities raised as an example in Section 7.2, and we sampled appropriately both at a stratified scale and in a random manner, we could extrapolate (extend with confidence) the results of our surveys to the wider area and suggest that this might be an overall representation of the current situation at the greater geographical area.

For instance, if the Large villages which were *actually* interviewed were found to accidentally trap an average of two dugongs a year, we might, with reasonable justification, suggest that *all* Large villages in the area

accidentally trapped an *estimated* 20 dugongs each year. We could do the same estimations for Medium and Small communities with similar reasonable degrees of confidence. But if we had only sampled Medium-sized villages we would never be in a position to say what occurred at Large or Small villages.

It is thus important that bias be eliminated from both Site-Level and Fisher-Level surveys so that the results can be extrapolated across much greater geographical areas than the project resources can address.

The Need for Clear, Explanatory Records – Maintaining records of who the project *did* and *did not* interview is as important as the interviews themselves. Similarly is a brief description of each community visited. A data analyst might wonder "I wonder why all fishers were more than 60 years-old?" But what if this was a community where all youngsters had gone away to the large village to study, or where fishing was a dying art? None of these things are errors, but they could introduce error into the analysis stage if they did not come with accompanying information.

It is important for the Field Staff to briefly describe each community they visit, and list the number of people they approached, and the number of people who agreed to participate in the surveys, and where possible the reasons why community members did not participate. They should record the time of day, the number of people available to be interviewed, and the range of expertise amongst those present. Records such as these will assist the data analysts at a later date put into context the results of the individual responses.

8.0 Field Data Collection and Control

This is one of the most important aspects of this project. It is imperative that data sets are properly numbered and tracked and linked, so that all information the project gathers can be easily linked into GIS packages, and none is lost due to incorrect handling.

8.1 Serial Numbers on Surveys

Each Survey form has a blank space at the top right corner of the first page (see graphic below) so that Project Manager(s) can write a sequential Serial Number on each form. If forms are provided and each is numbered, it is easy to track them all as they come back from the field. If they are not numbered it would be hard to determine if any were missing. Survey form Serial Numbers will also allow us to track the geographical and tabular data collected during each interview (see Sections 8.4 and 8.5).

It is strongly recommended that each project use their Country two-letter International Organisation for Standardization (ISO) code as a prefix to each survey form (e.g. PG for Papua New Guinea, TH for Thailand, MY for Malaysia, AU for Australia). This can be followed by a three digit number to identify each individual record. Three digits (000 to 999) provides each country with 1000 potential sheets – which should be more than enough for this project. Each country project should thus number Survey forms as (e.g.) AU001, AU002, AU003, AU004.... and so forth. A list of all world ISO codes: http://www.iso.org/iso/english_country_names_and_code_elements. If there were multiple projects in one country, an additional prefix or suffix could be added to identify these.

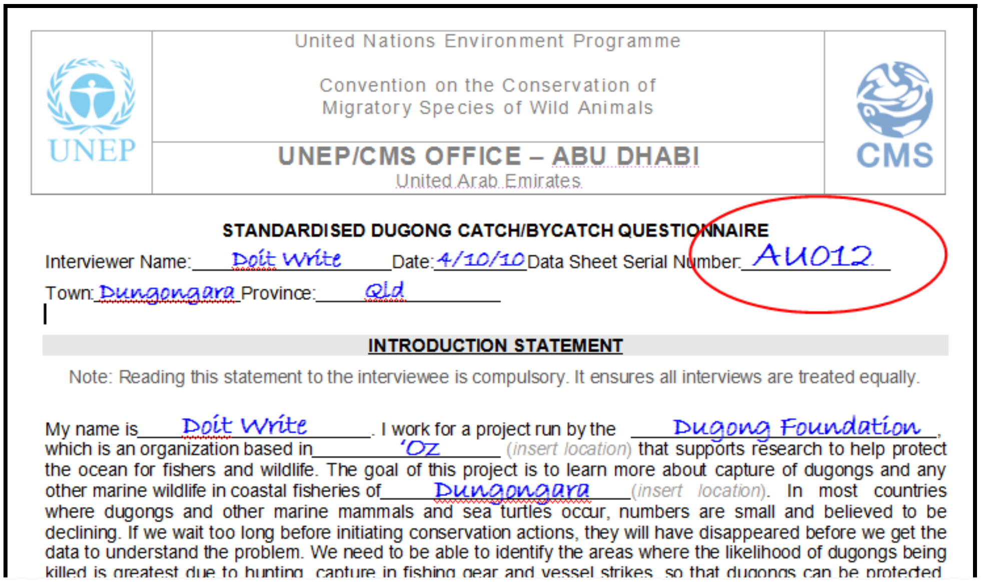

United Nations Environment Programme

Convention on the Conservation of Migratory Species of Wild Animals

UNEP/CMS OFFICE – ABU DHABI

United Arab Emirates

STANDARDISED DUGONG CATCH/BYCATCH QUESTIONNAIRE

Interviewer Name: Doit Write Date: 4/10/10 Data Sheet Serial Number: AU012

Town: Dungongara Province: Qld

INTRODUCTION STATEMENT

Note: Reading this statement to the interviewee is compulsory. It ensures all interviews are treated equally.

My name is Doit Write. I work for a project run by the Dugong Foundation, which is an organization based in 'Oz (insert location) that supports research to help protect the ocean for fishers and wildlife. The goal of this project is to learn more about capture of dugongs and any other marine wildlife in coastal fisheries of Dungongara (insert location). In most countries where dugongs and other marine mammals and sea turtles occur, numbers are small and believed to be declining. If we wait too long before initiating conservation actions, they will have disappeared before we get the data to understand the problem. We need to be able to identify the areas where the likelihood of dugongs being killed is greatest due to hunting, capture in fishing gear and vessel strikes, so that dugongs can be protected

8.2 Entering Data on the Table

The Table accompanying the Survey form is an integral part of the Survey. It allows the Field Staff to record individual sightings / strandings / events without having to have many spaces in the Form to record multiple events. The Table must be used every time an Interviewee indicates he / she has encountered dugongs / turtles / cetaceans. The Table should be completed as thoroughly as possible and is linked to the Survey form through the common Survey form Serial Number (referred to above in Section 8.1). In the graphic example below, note how the Survey ID Number in the left hand column links back to the survey ID number for the graphic example presented above.

In the example below, the Interviewee indicated that he / she encountered dugongs in 2009 and 2010, between March and April. Only one mother and calf pair was seen – look at row five of the data, where two dugongs were

recorded in deep water (D), one large and one small (L,S), during the day (D), and alive (A). A live (A), large (L) turtle was also seen (row six) in deep water (D), during the day (D). A wide range of data can be collated through this table, and linked to each individual Interview / Survey form. Notes can also be recorded in the far right hand column for data which does not fit any of these column headings. Field Staff should be trained and instructed to record information on this form as and how it is provided by the Interviewee, and ensure that each of the Sighting Record Numbers (column 2) are graphically recorded on appropriate charts / maps / graphics (see Section 8.3).

Survey ID Number	Sighting Record #	# / Type Individuals seen	Habitat	Size S,L	Mother - Calf Pair Y / N	Day / Night	Year	Month	Dead / Alive	Cause	Condition	Accidental / Direct	Reported Y / N	Notes
AU012	1	1 Dugong	S	L	N	D	'09	Mar	A	-	-	-	N	
AU012	2	1 Dugong	S	L	N	D	'09	Mar	A	-	-	-	N	
AU012	3	1 Dugong	S	L	N	D	09	Mar	A	-	-	-	N	
AU012	4	3 Dugong	S	LLL	N	D	'10	Mar	A	-	-	-	N	
AU012	5	2 Dugong	D	LS	Y	D	'10	Apr	A	-	-	-	N	
AU012	6	1 Turtle	D	L	N	D	'10	Apr	A	-	-	-	N	
AU012	7	1 Dugong	S	L	N	D	'10	Apr	A	-	-	-	N	
AU012	8	1Dug ong	S	L	N	D	'10	Mar	D	D	F	-	N	Missing one fin

8.3 Use of Graphics and Field Guides

"I recorded ten dugongs stranded in a net but I can't tell you where it happened". Can you imagine being on the receiving end of this statement? When we are trying to overlay threats over dugong / turtle / cetacean populations we need to know *where* these exist. To ensure that the project and data analysts can link the information which is gathered by the Field Staff when filling out the Survey forms and the Table to actual maps, it is ABSOLUTELY NECESSARY that each sighting / stranding / observation of a dugong or a turtle or a cetacean is marked on appropriate graphics, linked to the numbers in the Sighting Record column in the Table above. These graphics need to be pre-prepared, and context-sensitive. That is, there is no value in giving out maps of the East coast of an island when Field Staff are headed out to survey the West side of the island. This does mean a lot more work at the design stage for the Project Manager(s), because each set of numbered Survey forms will need to be accompanied by the relevant maps / charts.

Scale is another important factor when providing maps / charts / graphics for a particular area. If the scale is too fine (too much detail) a fisher might not

be able to describe the entire area he / she covers when out on a fishing expedition. Similarly, if the scale is too coarse (the graphic in each map covers a very large area) a fisher may not be able to put his / her fishing grounds into context. Imagine the following two scenarios when asking a fisher where he / she encountered a dugong:

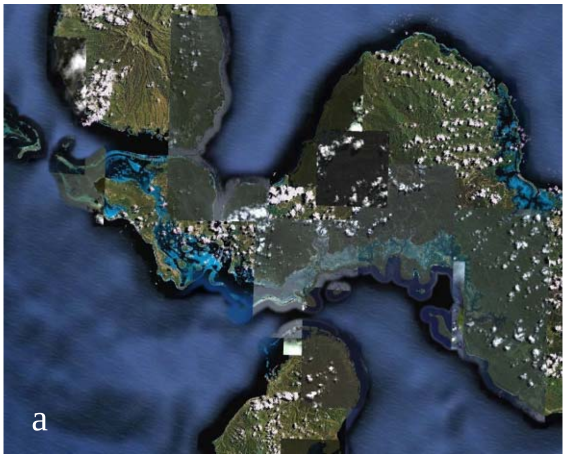

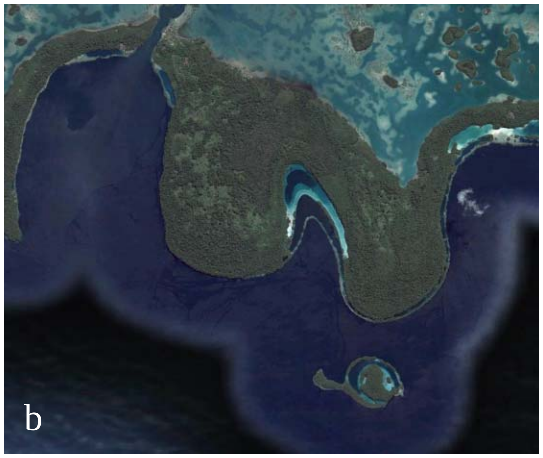

The right hand fine-scale graphic (b) is a zoomed-in graphic of the little island located slightly lower from the middle on the right hand-side of the coarse-scale graphic (a). Here we call a coarse-scale graphic one which covers a large area but in little detail, and a fine-scale graphic one that covers a small area but in greater detail. It is possible a fisher may not recognize his / her island in graphic (a) if he / she only paddles a canoe out from the beach, and thus a graphic on the scale of (b) would be more appropriate. However, a fisher might know that further West along the coast there is a village on a larger island where dugongs are taken regularly, and may be able to point this out on the coarse-scale graphic. It is therefore important for Field Staff to be provided with maps / charts / graphics of a variety of scales and relevant to the area in question *before* they set out to conduct interviews. These are *not* the sort of thing a Field Staff member could make up on the go. An example of graphics which might be supplied is presented in Annex II. One useful way to ensure you have adequate maps available is to print the maps in various scales and have them in a multiple-compartment folder, and then use them one you find out the best scale at which the fisher can provide reliable and useful data.

There are also a variety of choices when choosing maps and graphics. You might have road maps available to you, or you might have detailed cartographic maps, or have access to Google Earth or Google Maps. Any one of these are suitable, so long as they clearly show areas which are relevant to the people being interviewed. We have found that prints of the Google Earth graphics tend to come out too dark, and that colour maps where the sea is blue are much better understood by fishers not used to reading maps.

In the end, there are really only three key characters of maps / charts / graphics that you need to take into consideration: (1) they must be of the right place; (2) they must be at the right scale, and (3) that they are clear enough for fishers to read. We recommend you have one or two large maps at a small scale so that a large area can be seen, and then a series of smaller maps at a large scale in which a lot of detail can be noted. Precisely which type of graphic you use is up to you.

Field Guides – Appropriate field guides of the animals in question are the other important set of documents the Field Staff need to have available so that they can show them to the Interviewee when asking questions about species. Annex I shows a sample field identification guide for dolphins, turtles and dugongs, which is a good example of the material your staff will need, and also another just dealing with turtles from the Queensland government in Australia. You might also want to use a seagrass guide (see Annex II for an example) so that you can find out what seagrasses are found, and to show fishers what you mean by seagrasses. It is recommended these guides are laminated (plastic covered) as they will likely be handled by a number of people and may also get wet. A variety of similar guides are available and it is up to the project management to make these available to Field Staff prior to embarking on interview sessions.

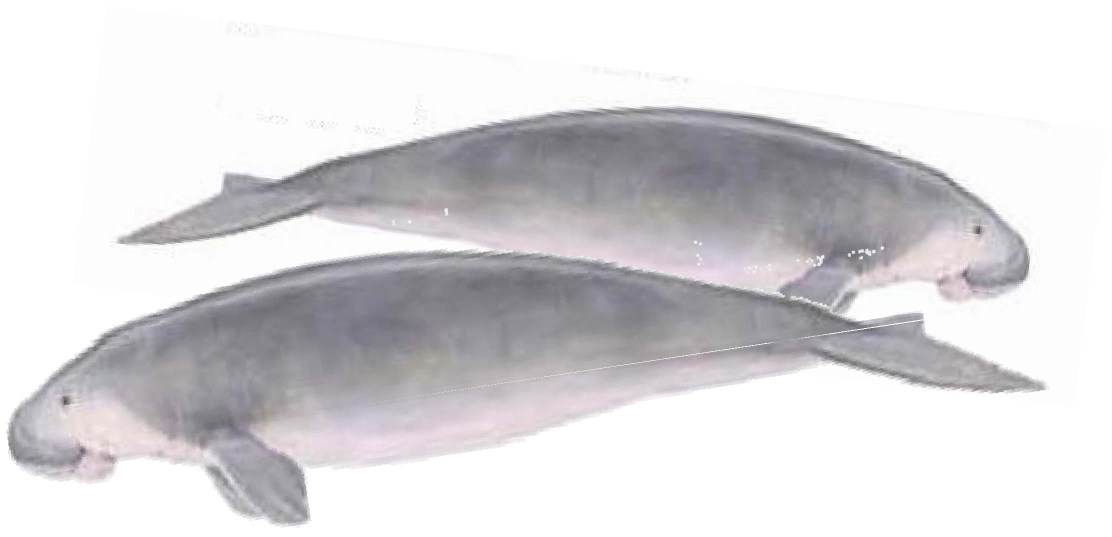

8.4 Linking Graphics to Table Data to Survey Numbers

All data collected by this project must be interlinked. Data from Tables must be linked to Survey forms, and data from graphics must be linked to Tables and Survey forms. It is important that the Field Staff know how to label each graphic sheet so that they become a part of an individual interview. In the graphic example below, please note how the graphic itself is linked to one particular Survey Form, number AU012 (circled in red). All graphics need to be labeled in this manner. There needs to be at least one map PER interview, with all the fishing areas, seagrass areas and dugong/turtle/mammal sightings / strandings annotated on it. This can then be uploaded into Google Earth and emailed to us for later analysis.

Once a graphic is linked to a particular Survey Form (in this example to form AU012), all annotations which are made on that graphic can be geographically linked to that form also. For instance, information on the areas where the person fishes (indicated by the white-lined areas in the graphic below) can digitized at a later data (the easiest way is using Google Earth as described below in Section XX) so that all fishing locations or seagrass areas can be established for the particular community.

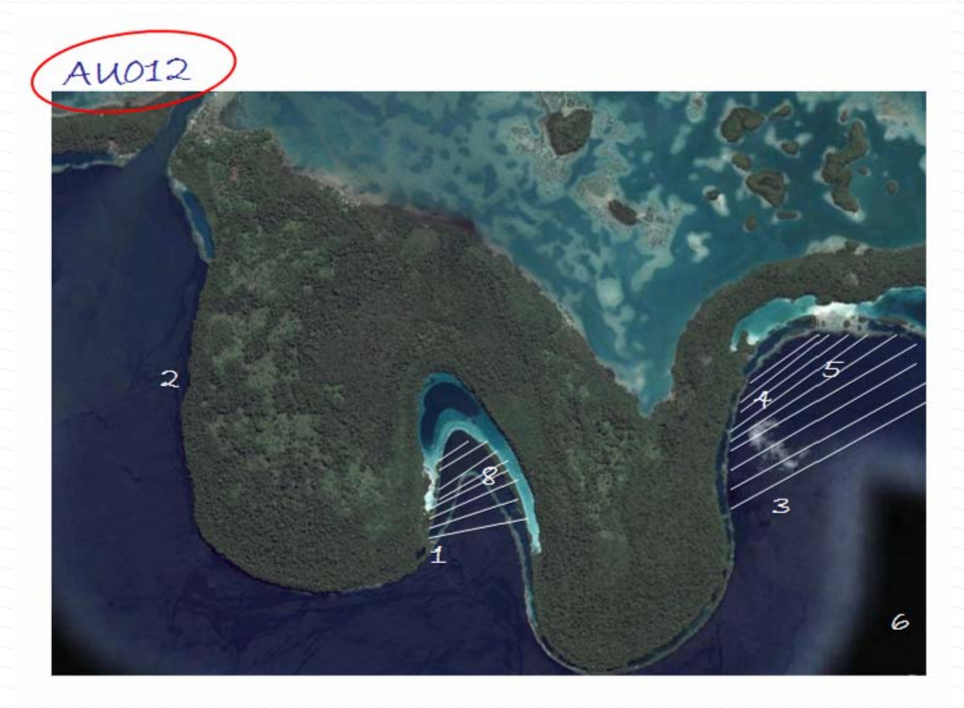

During the interview session, the Interviewee will ask the fisher where he / she normally fishes (see Question 19), and ask that these areas be drawn-in on a map. They can be put up as shaded areas, or lines, or whatever the fisher is able to do. Some guidance may be needed to help the fisher

understand where each place in the map refers to (again requiring some background knowledge of the area prior to conducting the interview – such as names of places, notable bays and estuaries, deep water areas, reef areas, etc). All kinds of fishery data can be drawn in on the graphic: You could record one type of fishing gear use in one colour, another type of fishing gear in a different colour. Or with a different pattern. You could record high season and low season fishing areas. An example of how this could be done is shown in the graphic below (circled in red)

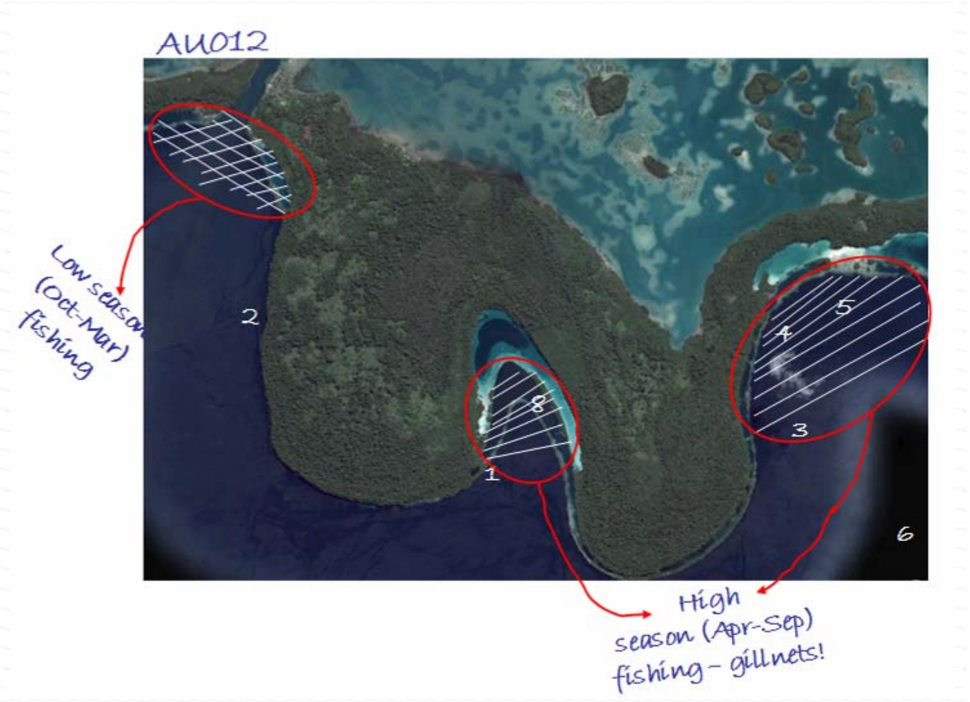

In addition to this, all actual sightings of dugongs / turtles / cetaceans can also be displayed on the graphic. If you check back to the example of the completed (sample) Table in Section 8.2, you will recall that the Interviewee had highlighted eight unique encounters (numbered 1-8 in the Sighting Record column). Each of these sightings had a suite of information related to them: the number of individuals, day or night, calving pairs, dead or alive, that sort of thing. That information can be graphically linked to the Survey by simply recording each number clearly on the relevant graphic. Obviously the more accurately the fisher is able to mark these, the better results you will be able to collect for the overall Survey, but in general what the project is after are the general distribution of dugongs / turtles / cetaceans, not the exact position of each one (especially as they will have moved by then!), so a rough approximation is acceptable. Note in the graphic below how each of those eight individual sightings is recorded (circled in red). These points can be digitized and the attributes of each (what was actually seen at those locations) can also be entered into GIS layers for subsequent risk analysis.

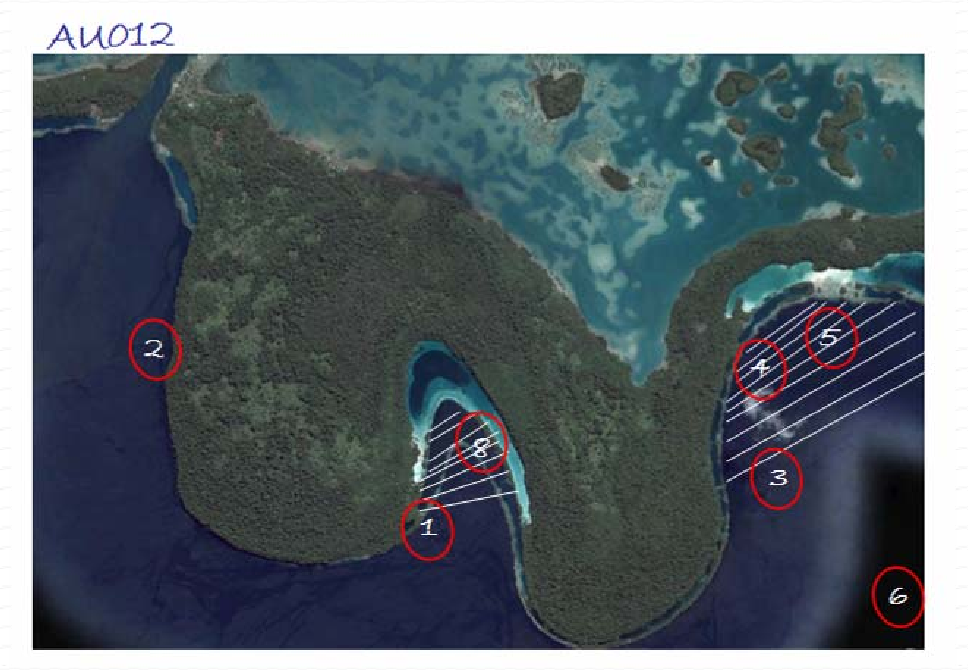

You can see, then, that all data collected during an interview can be drawn on a map or a chart or a Google Earth image, and this can be linked to the Table in the Survey form, which can be linked to the information in the Survey itself. Field Staff should be trained to record these data sets and keep graphics together with individual Survey forms, for subsequent analysis.

A key point to remember here: Use a new graphic for each interview! If you already have data marked on a sheet that you use in a new interview, you could introduce bias in the same way as a leading question could, and we learnt in Section 7.3 that this is not a good thing. A fisher might be tempted to just say he /she fishes in the areas already highlighted, rather than describe the particulars of his / her fishing habits, for instance. It is probably also important to note at this stage that each field outing will require a large number of sets of maps and charts so that these can be used one at a time. There should be one set of graphics for each Survey – put another way, each set of individual answers (data) should be recorded on a different sheet.

8.5 Training Requirements

Clearly then, there is a requirement for a substantial amount of training for the Field staff. It is not possible to rapidly introduce the objectives of the project, give them the Survey forms, and send them on their way. Each Interviewer will need a set of basic skills in order to return with acceptable and valid results from the interview surveys.

Project Objectives – Field staff will need an introduction to the purpose of the project, what it aims to achieve, and how. During this session Field Staff need to be told about ethical issues (such as explaining the consequences of participating in the survey), and about intended outcomes of the work (estimates of population size and key threats, and ultimately a risk analysis that shows key conservation areas in a graphical format). Field Staff should also at this stage receive instruction on data integrity, and the role they as individuals play in a greater process – how the data will be used to develop National priorities and ultimately Regional priorities.

Interview Techniques – What has been mentioned in previous sections about interview techniques is crucial background information for Field Staff. It is the responsibility of the Project Manager(s) to make sure that the Field Staff have an understanding of the basic concepts of personal interviews and approaches.

Practice the Interview – It is essential that the field staff, those who will conduct the interviews, practice amongst themselves and with several 'trial' volunteers several times (at least ten) before going out to the field to get a good understanding of how to ask the questions and how to conduct the interviews. This is a very important aspect of preparation.

Sampling Design and Bias – Field Staff need to understand all about bias and sampling design as they will be out in the field and will need to make independent decisions related to who they interview, how many people they interview, and how to adapt when things do not go as planned (for instance, if they arrive at a site and find out that the fishers have temporarily moved). Preferentially, Field Staff should be involved at the project design stage so that they understand why certain communities were chosen for survey while others were not.

Dugong Biology and Identification – A basic understanding of what a dugong is and its life history are critical for the Field Staff to be able to ask the right questions at the right times. As noted above, the Interviewer does not want to be the subject of ridicule for not knowing about the subject he / she is asking about.

Turtle Biology and Identification – Similarly, a very basic understanding of turtle identification (how to tell species apart) and biology / life history should be part of the up-front training. When a fisher is unsure of the species of a turtle, it is the guidance from the Interviewer that might provide clarity.

Completing the Forms and Maps – Finally, Field Staff need to be shown how to complete the forms and maps competently and accurately. Imagine the

waste of resources if the Field Staff did not return with linked tables and graphics and forms and data analysts were unable to piece together the pieces of the puzzle. During the training session we recommend that the Project Manager(s) run through the questionnaire process several times, and then have the Field Staff trial the Forms amongst themselves and maybe even with friends or colleagues. We can not overstate the value of trials before going out into the field!

8.6 Miscellaneous Questionnaire Details

The Survey Questionnaire is long, admittedly, but it is designed to capture a large range of data and to make sure that the results are statistically valid and robust. Some key points to remember about the Survey are as follows:

The Value of a Blank – Not all questions need to be asked all the time. If an Interviewee says he / she last saw a dugong five years ago, it would not be appropriate to ask the next question: "How many did you see last year?" However, when a question is not asked, it is important that this be recorded (the Survey Document has tick boxes on the left side for just this purpose), so that when analysing the data one can be sure the question was not inadvertently omitted. This is particularly relevant several months after interviews have been conducted and the memory of the interview session has faded.

The value of "I don't know" – Many surveys do not take into account the fact that someone may not know something. It is extremely possible that a fisher does not know if there are dugong experts in nearby villages, but it would be unrealistic to think he / she might not know this for his / her village. The Survey form allows an 'I don't know' answer in most of the questions, to accommodate this type of response. It is up to the Interviewer to clearly determine that the Interviewee is not aware of something, and check the answer as 'Don't know'.

Questions to Determine Reliability – The Survey form has a number of questions to determine Interviewee response reliability. You might think they are repetitive or redundant, but their inclusion is on purpose. For instance, if a respondent says he /she knows what a dugong is, then it is likely they can describe some simple differences between a dugong and a dolphin (such as the shape of the head, as an obvious example). It is less likely however that many of them could tell how long a dugong lives, unless they have participated in previous surveys / discussions on dugongs. Questions such as these are designed to judge respondents' honesty and accuracy, and thus are an important component of the survey, regardless of how different they may seem.

Interviewee Reliability – Questions 102-107 are confidential questions to be answered by the Interviewer and which relate to how comfortable and accurate the Interviewee's responses were. For instance, a respondent might say he / she has never caught a dugong, but there may be some underlying reason for the response, such as fear or reprisals or concern about how the data will be used. Similarly another respondent might say they have caught over 100 dugongs but this could be simply an exaggeration rather then factual. It is important that these feelings are captured for each survey so that data analysts in the future can determine reliability of individual responses – particularly when uncharacteristic responses are provided.

9.0 Uploading Graphics and Spatial Data

By now you will have realized that the graphics data collected by this project is one of the three key aspects of the project. In order to make the graphics data available for analysis, and so that they can easily be shared and transmitted around the planet to others, they need to be in an electronic format which can easily by sent across the internet, and which can be imported into more sophisticated analysis packages. The easiest way to do this is to load them into the freely-available software Google Earth, (which you probably already have!). You do not need the professional version for this: the freeware version will be more than sufficient. The latest version of Google Earth can be downloaded for free at http://www.google.com/earth/index.html.

9.1 Creating Google Earth Layers

We are sure you are already somewhat familiar with Google Earth, so we won't get in to how you install it and use it. For what is needed in this step, you need to create some folders in which to store your data. In Google Earth right click on **My Places** at the top left of your screen and click on **Add**, then **Folder**, and add three new folders. To do this, make sure you highlight the **My Places** folder, then right click the mouse and follow the links to **Add** and then **Folder**. In the pop-up window that appears, create three new folders called **All_Sightings**, **All_Fishing_Areas** and **All_Seagrass_Area**s. Once you do these simple tasks you are ready to enter all of your project data.

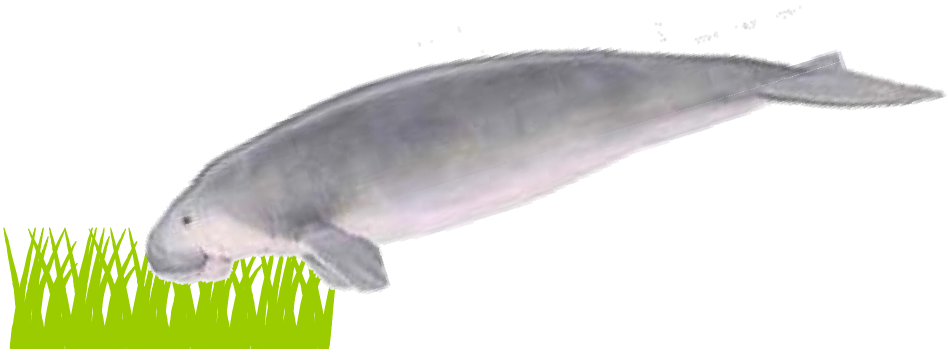

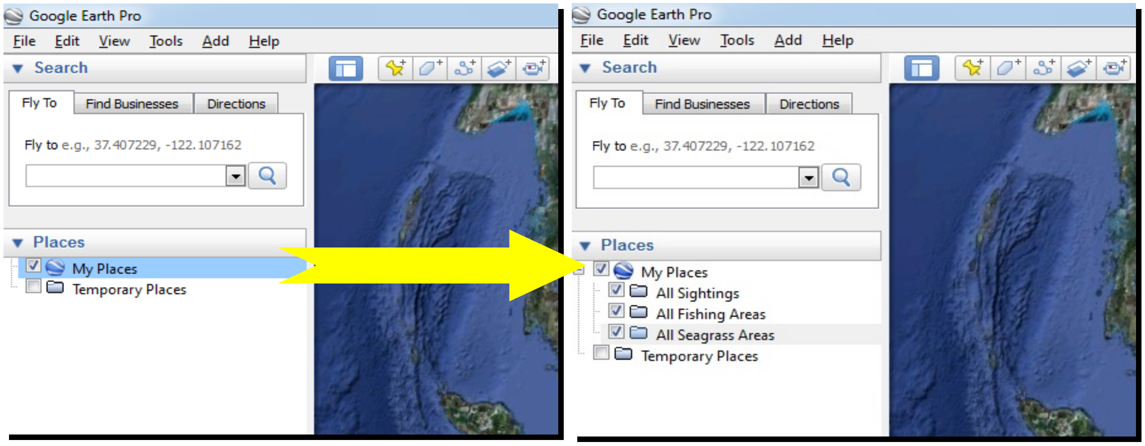

9.2 Adding Sighting / Stranding Records

Data can come in various types, and sighting or stranding records are called *Point* data, because they get shown as a point on a map. Each of these *Points* can have a number of descriptors, or *attributes* as the computer people call them, which describe what the point represents. Points obviously have location descriptors (latitude and longitude) but can also have the species, the size, the fate and that sort of thing associated with them. **Placemarks** are a simple way to save point locations in Google Earth. Dugong sightings and strandings identified by survey participants can be mapped in Google Earth using **Placemarks**.

To do this, highlight the '**All_Sightings**' folder you created earlier by clicking on it and then press the **Placemarks** button on the Toolbar to add a **Placemark**, or data point for each individual sighting record and / or stranding record identified in each questionnaire. When you click on the **Placemarks** button you will get a pop-up window in which you can enter the details of the specific record, and an icon that normally looks like this which you can move around until it is in the same place on the Google Earth map as it was on the graphic from the questionnaire. All you have to do is look at the graphic completed by the fishermen, select the same area in Google Earth, and insert the **Placemark**. To move the icon around, just click and hold and move it around with the mouse. Zoom in as required to place the icon as accurately as possible. The sightings should be named (in the **Name** field at the top of the pop-up window) according to the self-generated **Individual Record ID** in the Sighting Records table – this might look something like **MRF003-2** or **MY012-14**. Please refer to the Sighting Records worksheet in the Excel File containing the data from the questionnaire data sheets to find this unique record number – by keeping this record number in Google Earth you maintain the link back to the original respondent all the way through the process.

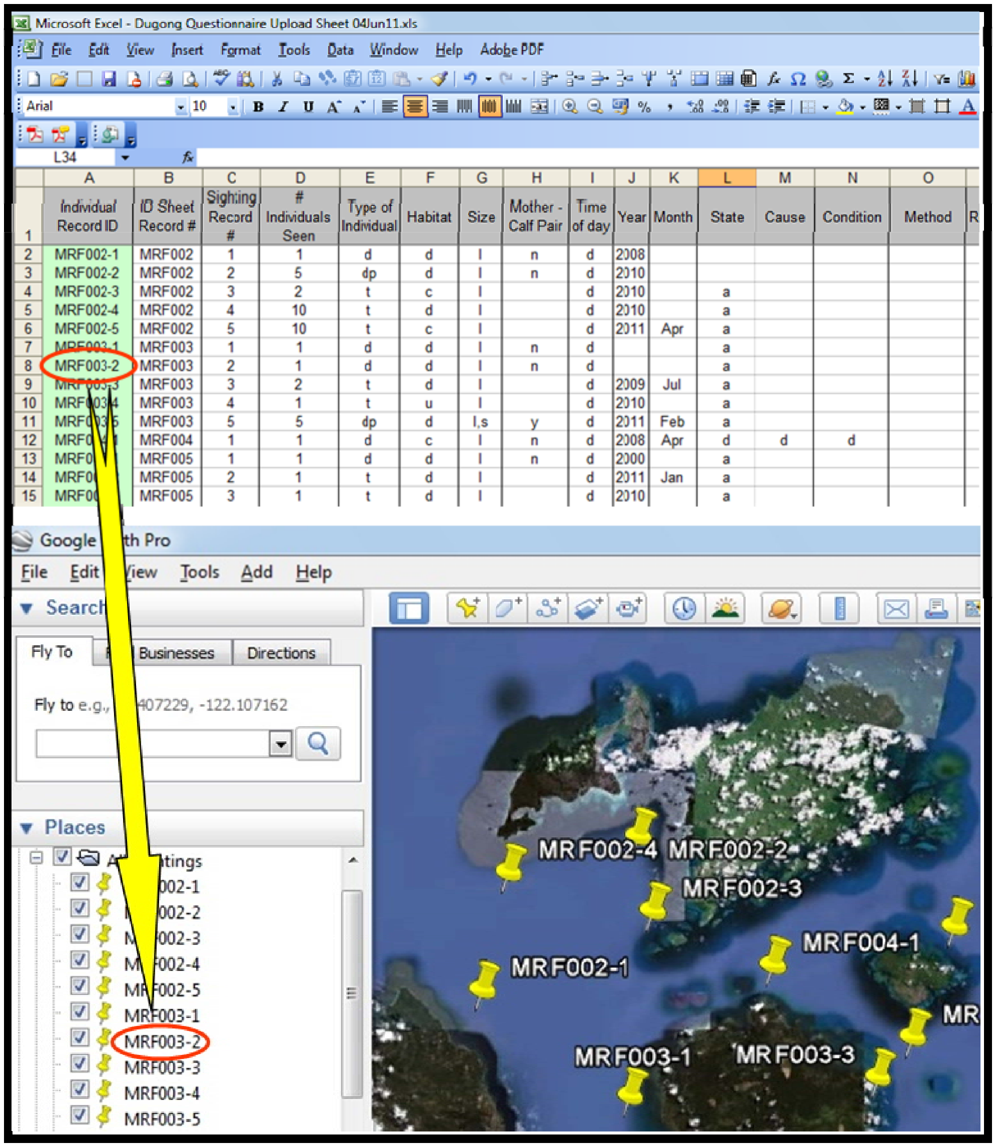

9.3 Adding Fishing and Seagrass Areas

The other type of data you will be dealing with is **Polygon** data (which covers an area, rather than just a point). This data could include such data as a patch of ocean where a fisherman fishes, or a bay where he knows there is seagrass. You can draw **Polygons** in Google Earth to map where fishing and seagrass areas were identified in each particular questionnaire. To do this, highlight the **All_Fishing_Areas** or **All_Seagrass_Areas** folder by clicking on it. Then click the **Polygon** button on the Toolbar to draw a polygon. Again, look at the graphic drawn by the fisherman, select the same area in Google Earth, and create a polygon by moving your mouse and cursor over the screen to map fishing or seagrass areas clicking on each extent of the polygon. As you go clicking around the outer points, you will see a polygon

developing on the screen. You can change the way your polygons appear on the screen by highlighting the features name (e.g. **MRFOODFAa**) by clicking on it, then right click and scroll down to 'Properties'. Select 'Properties' and then click on the 'Style, Color' tab. Further information on doing all of this can be found in the Help section of Google Earth.

The fishing area names should be made up of the Questionnaire ID Code, the letters FA, and a consecutive letter in cases where there are more than one layer per questionnaire – such as when a fishermen indicates two main fishing areas (e.g **MRF001FA**, **MRF002FAa**, **MRF002FAb**, etc., where MRF002 is the questionnaire ID sheet, FA stands for Fishing Area, and the a or b suffix is to denote each different area marked on the sheet).

Seagrass areas should be similarly named with the code SA using the same name format as fishing areas (e.g **MRF002SA**, **MRF003SA** etc.).

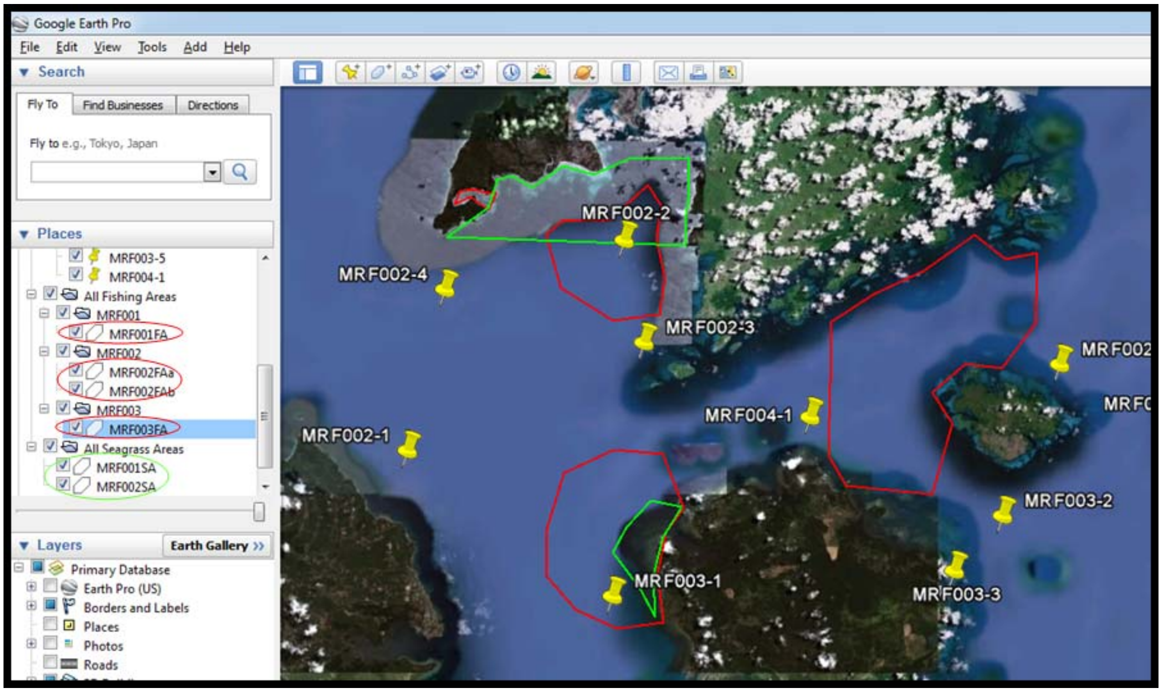

9.4 Saving our Google Earth Files in .kmz Format

Of course, the idea of entering all of this data is so that is can be analysed using sophisticated GIS analysis programmes. A GIS programme is a system for storing, analysing and managing data which are spatially referenced to the earth. GIS is a type of technology that is used to make maps, and store information about locations, points or areas in a computer. There are many GIS software options available. In this training manual we use ArcInfo by a company called ESRI, but there are several other options available.

For the files to be easily imported into GIS packages, it is preferable that they are saved in Google Earth as **.kmz** files. You will need to create some new directories / folders on your computer in which to save your files and folders, somewhere that will be easy to find and work with. First, create a master directory such as **C:\My Documents\Dugong_Project_Data**. Inside this directory, create another folder called **All_kmz_data** because you will have lots **.kmz** data to keep track of later on! Inside the All_kmz_data folder, create two more: One called **Individual_FA**, and one called **Individual_SA**. Your file structure should look something like this:

C:\My Documents\Dugong_Project_Data

C:\My Documents\Dugong_Project_Data\All_kmz_data

C:\My Documents\Dugong_Project_Data\All_kmz_data\Individual_FA

C:\My Documents\Dugong_Project_Data\All_kmz_data\Individual_SA

9.4.1 Saving Sightings data in .kmz Format

For this first part, you simply need to create a single **.kmz** file to save all the Sightings data. Once all your point data is entered, right-click the '**All_Sightings'** folder in Google Earth, and then click on **Save Place As**. In the pop-up **Save Place As** window, browse until you find the directory / folder you earlier created for this project in the left hand panel. Then select **.kmz** in the **Save as Type** field just under the **File name** field, keep the file name as 'All_Sightings' and click **Save**. It's that easy - your sighting data are now saved in **.kmz** format and ready for subsequent import into a GIS package.

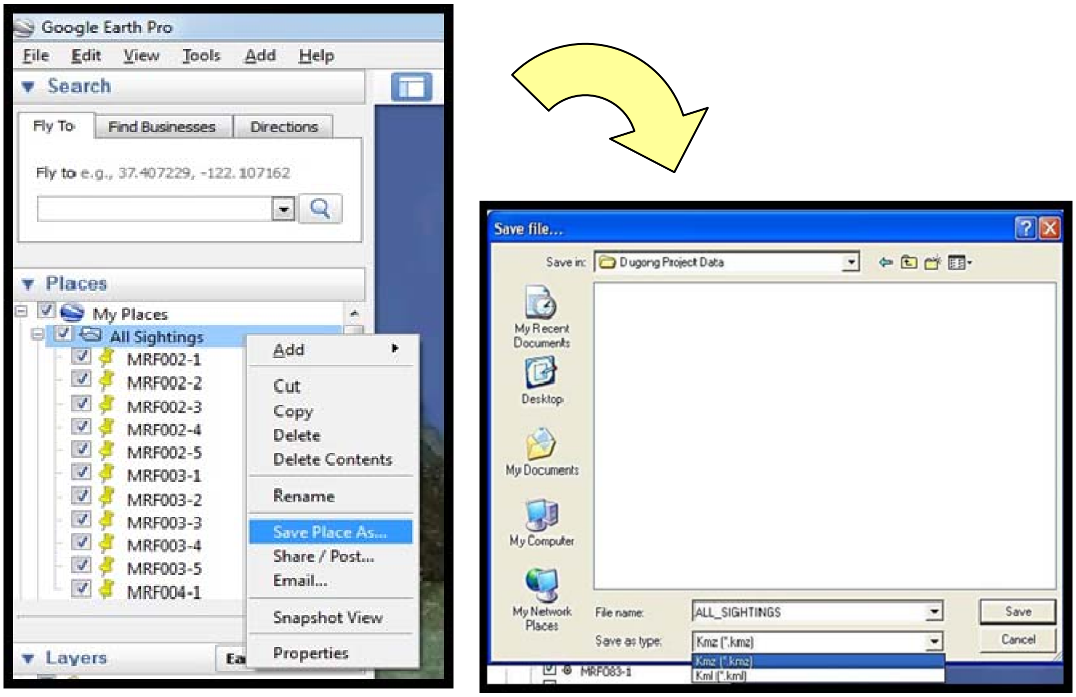

9.4.2 Saving Individual Fishing and Seagrass Areas as .kmz Files

Earlier you created a **.kmz** file which included all the point data. It is perfectly ok to save all these in one single layer because they will show up and be used as one layer on a map later on. But with fishing and seagrass data we will want to do additional analysis so that we can find out where overall distribution of seagrasses is, and where the highest density of fishing occurs.

Keep in mind, when two fishermen report the location of seagrass areas these might overlap. But this does not mean there is more seagrass in the areas where they overlap, it just means both fishermen know about the place. But with fishing areas, if both fishermen overlap in their fishing areas, it means there is double the fishing pressure in the overlap region.

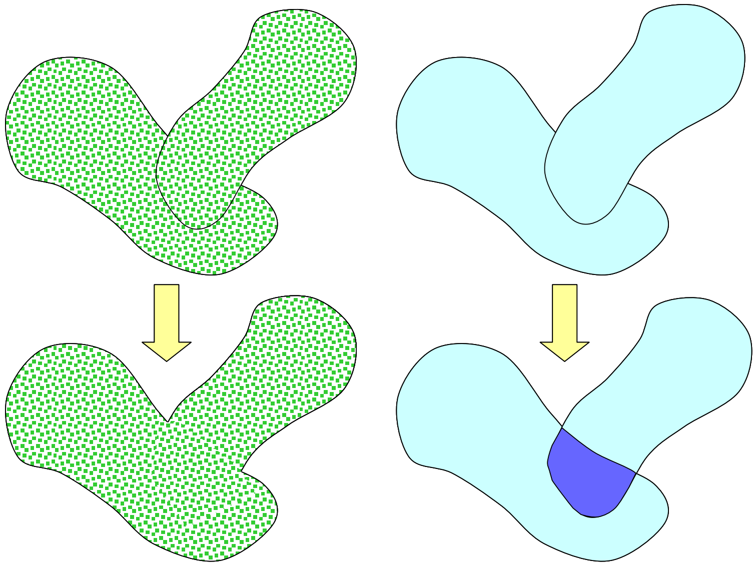

Seagrass reported twice is still just seagrass.
Fishing reported twice is double fishing and thus an overlap.

It is important to know this because later on these layers will be treated in different ways: seagrass areas will be added together to make one large layer, and fishing areas will be overlapped to look at density hotspots. In order to distinguish individual fishing and seagrass areas, we need to save each fishing / seagrass polygon as its own individual file in Google Earth.

All you have to do in this step is save each polygon one at a time the same way you did above with the **All_Sightings** data, but here you have to save each individual polygon as a new **.kmz** file and keep them organised in their respective folders that is, All Seagrass Areas must be saved individually into the **Individual_SA** folder and all Fishing Areas need to be saved individually into the **Individual_FA** folder. You need to make sure you keep the ID names consistent as you save files one at a time so that each file can be traced

directly back to each respondent. To do this, right-click on the polygon name you would like to save (e.g. **MRF002FA**), and then click on **Save Place As**. In the **Save Place As** pop-up window, browse to the **Individual_FA** folder, and save the file with the same name but in **.kmz** format. Repeat this procedure for the all the Seagrass polygons and all the Fishing Area polygons you created in Google Earth. This file-saving process is not very different to what you did above for point data, but it can get a little long and tedious when you have many polygons!

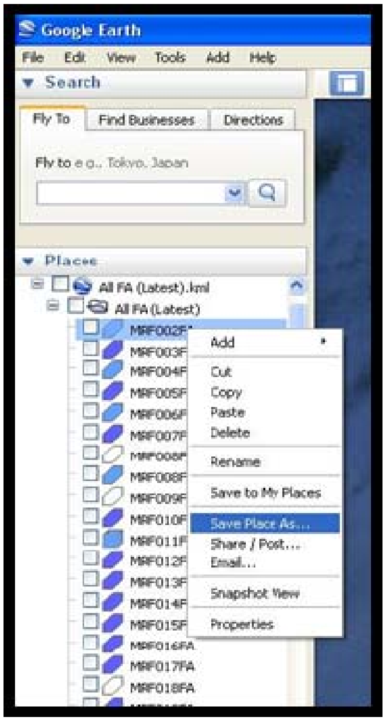

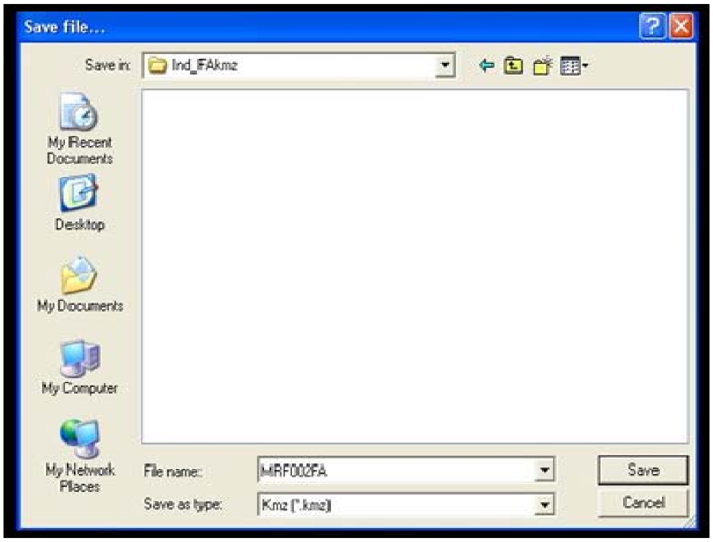

This is far as you need to go, and if you've done all of that then you've done well. Your point data and your polygon data should now be all ready for analysis. The next chapter is going to explain how to convert these files into a format that can be manipulated to illustrate your findings using GIS. This can get a little tedious so it would be good to have a little help from someone who is handy with GIS programs.

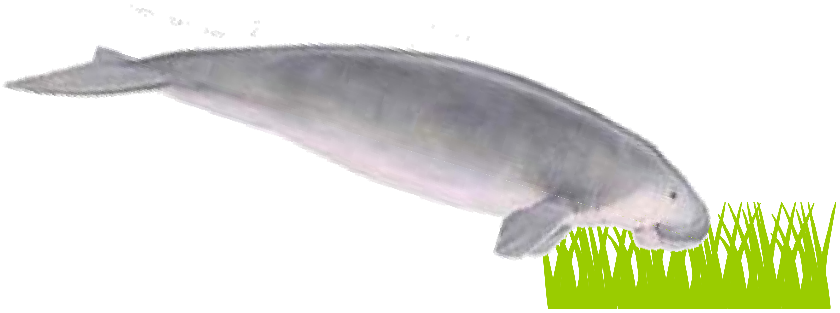

10.0 Literature Cited and Additional References

Aragones L.V., Jefferson T.A., Marsh H, 1997. Marine Mammal Survey Techniques Applicable in Developing Countries. Asian Marine Biology 14 15 – 39.

Fowler Jr., F.J. 2009. Survey Research Methods, IVth ed. Sage Publications, Thousand Oaks, CA.

Husar S.L. 1978 *Dugong dugon*. Mammalian Species 88: 1 – 7.

Lawler I., Marsh H., McDonald B., Stokes T. 2002 Dugongs in the Great Barrier Reef: Current State of Knowledge. In: Centre CRR (ed)

Marsh H. 1997 Going, Going, Dugong Nature Australia pp 51 – 57.

Marsh H. 2008 *Dugong dugon*. IUCN Red List of Threatened Species.

Marsh H., Lefebvre L.W. 1994 Sirenian Status and Conservation Efforts. Aquatic Mammals 20 155 – 170.

Perrin W.F., Reeves R.R., Dolar M.L.L., Jefferson T.A., Marsh H., Wang J.Y., Estacian T.A. 2002. Report of the Second Workshop on the Biology and Conservation of Small Cetaceans and Dugongs of South-East Asia. Siliman University, Dumaguete City, Philippines

Poonian C.N.S., Hauzer M.D., Ben Allaoui A., Cox T.M., Moore J.E., Read A.J., Lewison R.L., Crowder L.B. (2009) Rapid Assessment of Sea Turtle and Marine Mammal Bycatch in the Union of the Comoros.

UNEP (2002) Dugong Status Report and Action Plans for Countries and Territories.

Annex I – Sample Field Guides

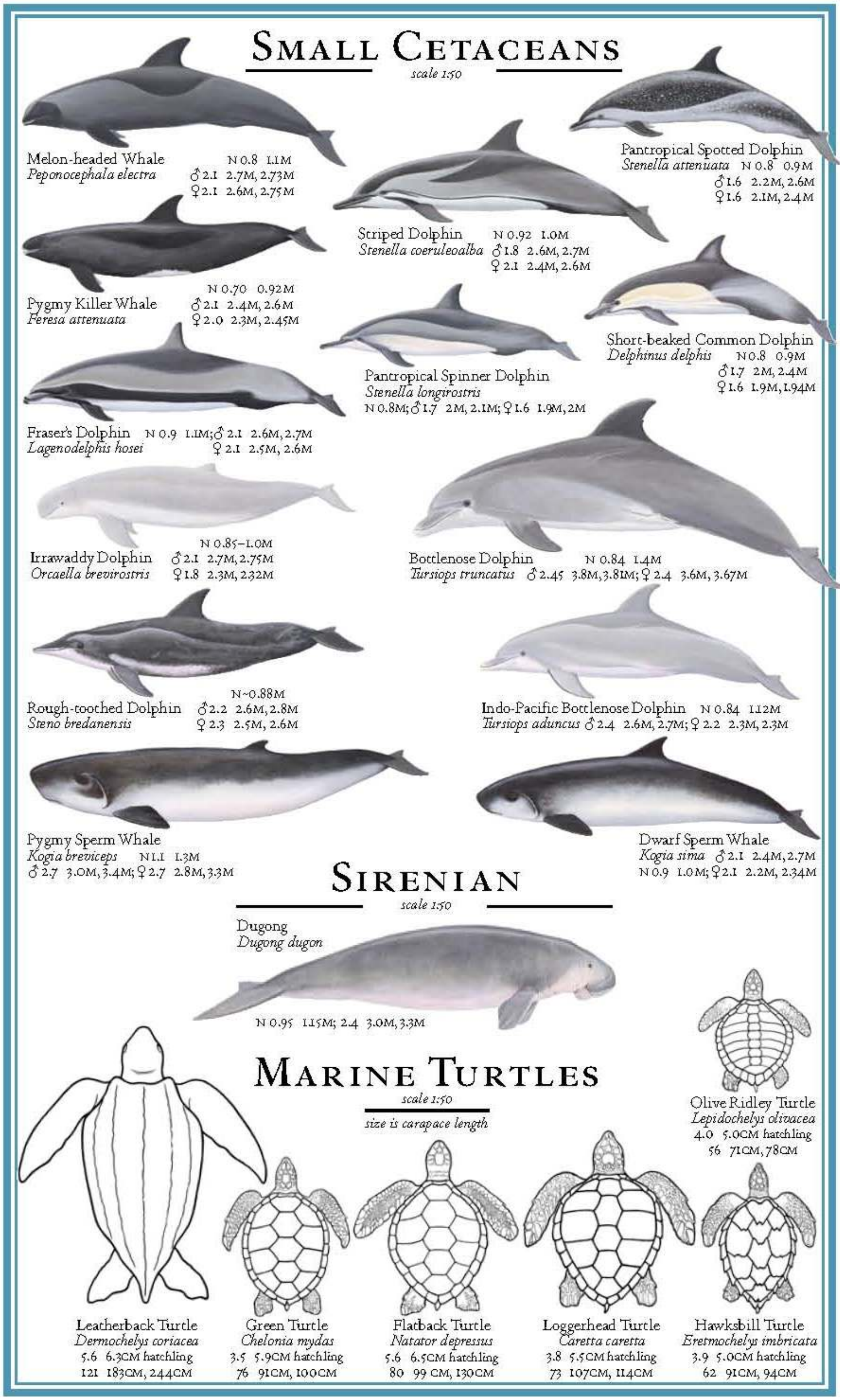

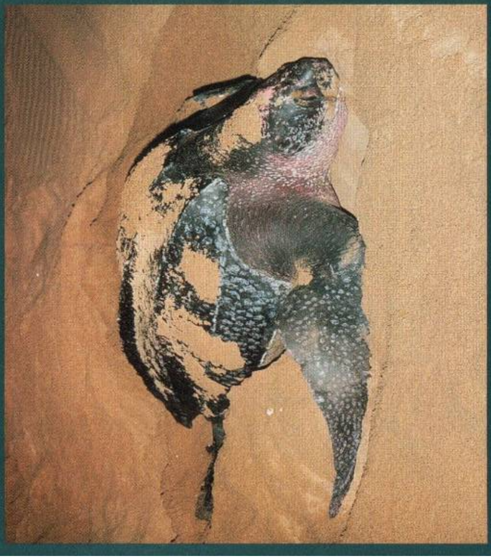

Dermochelys coriacea (Leatherback turtle)

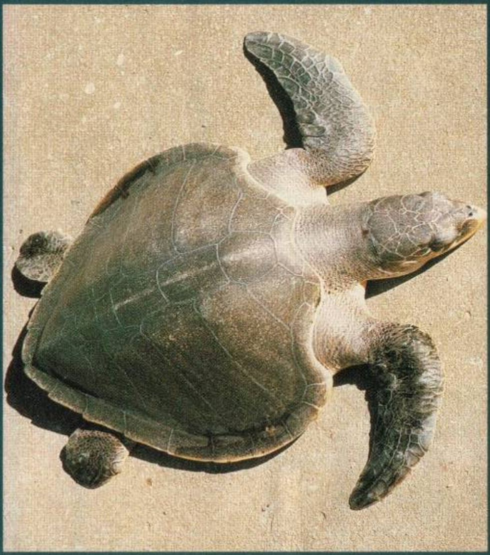

Lepidochelys olivacea (Olive ridley turtle)

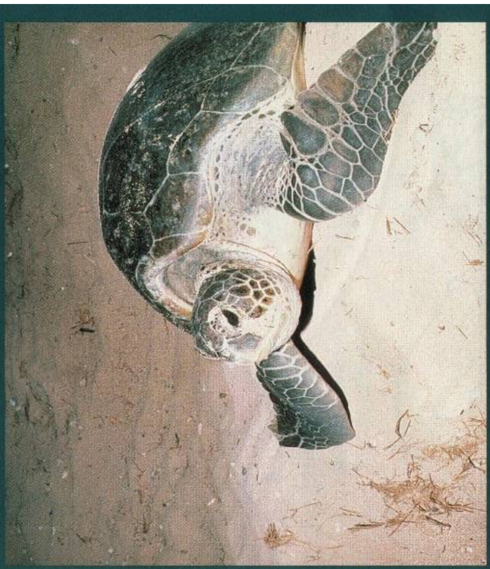

Chelonia mydas (Green turtle)

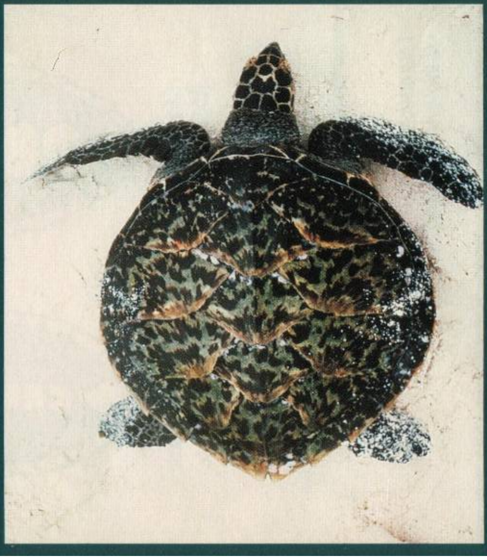

Eretmochelys imbricata (Hawksbill turtle)

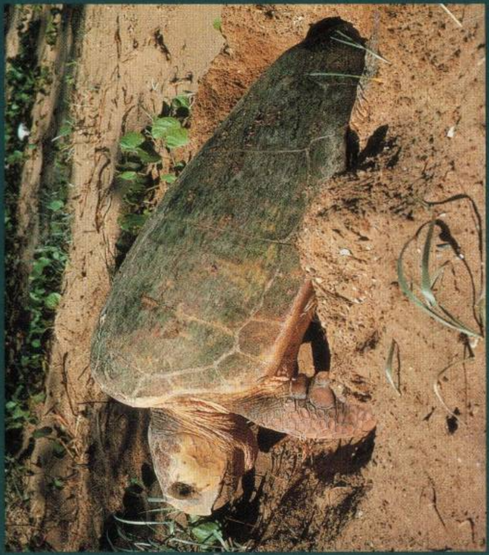

Caretta caretta (Loggerhead turtle)

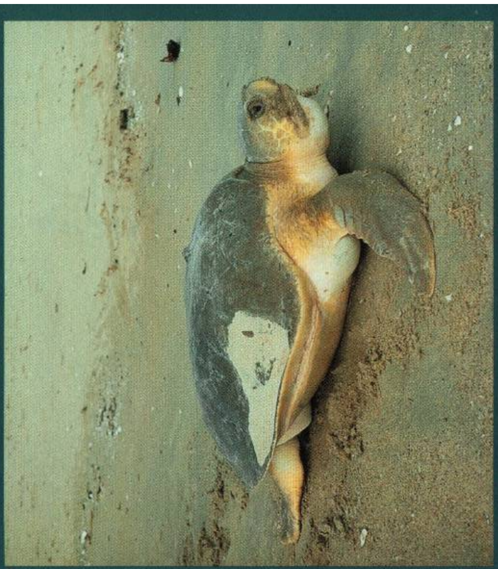

Natator depressus (Flatback turtle)

Annex II – Sample Seagrass Field Guide

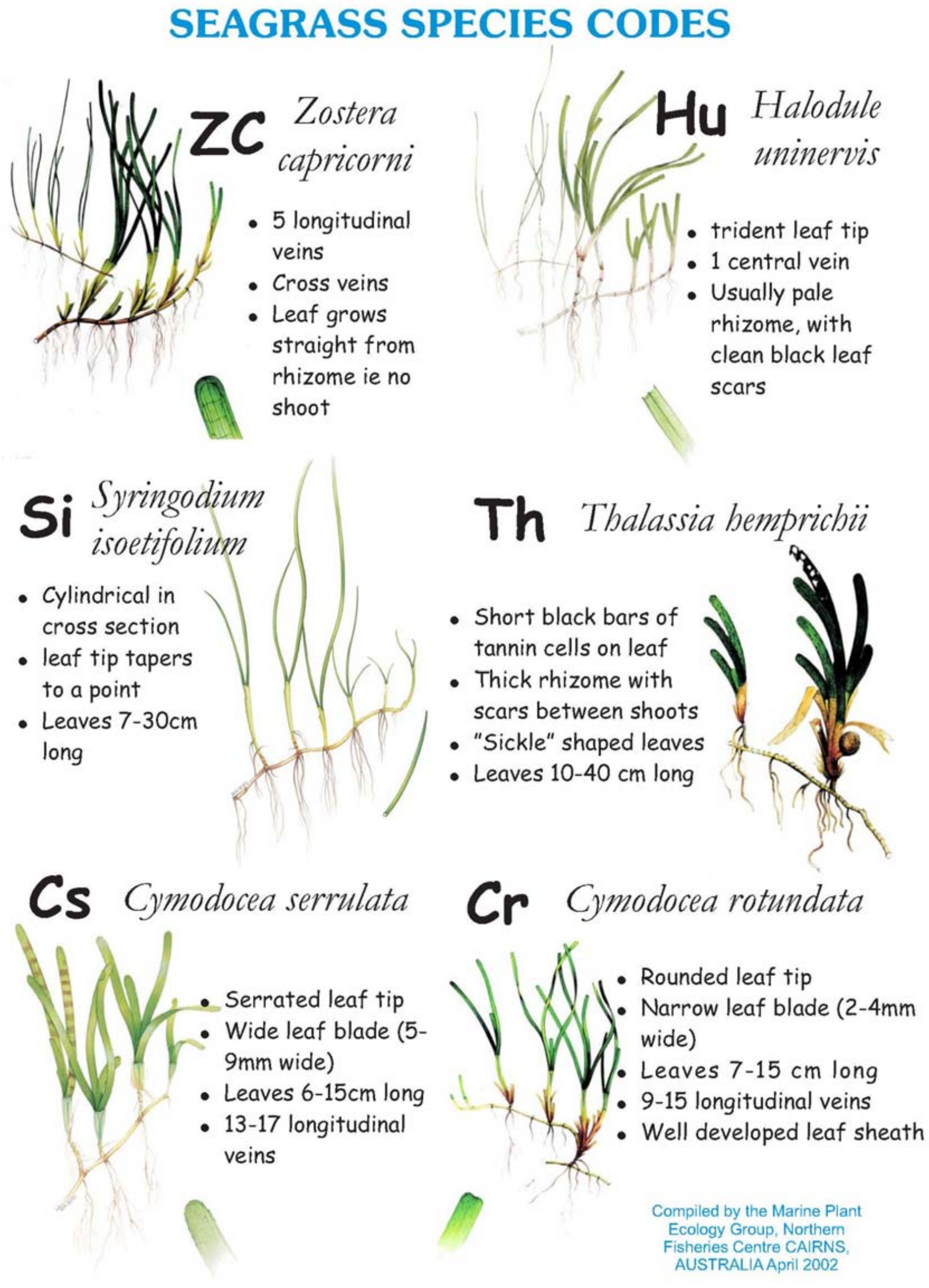

SEAGRASS SPECIES CODES

Ho *Halophila ovalis*

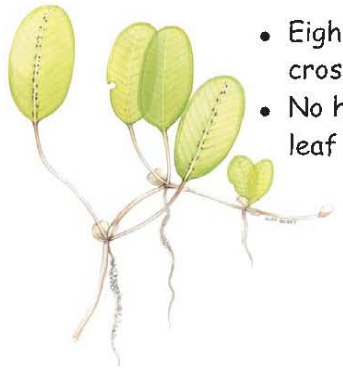

- Eight or more cross veins
- No hairs on leaf surface

Hs *Halophila spinulosa*

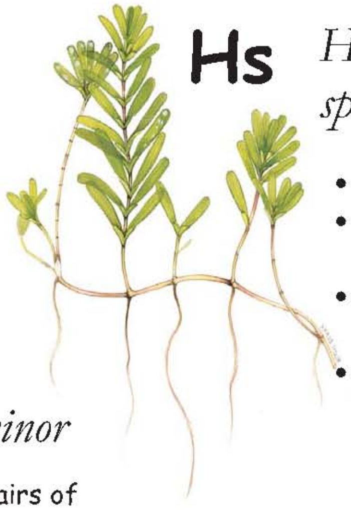

- Fern like
- Leaves arranged in opposite pairs
- Erect shoot up to 15cm long
- Found at subtidal depths

Hm *Halophila minor*

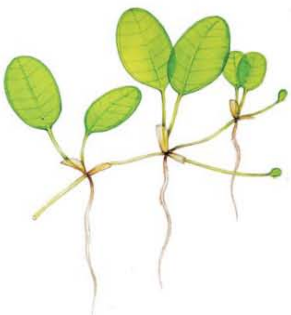

- Less than 8 pairs of cross veins
- Small oval leaf blade

Ht *Halophila tricostata*

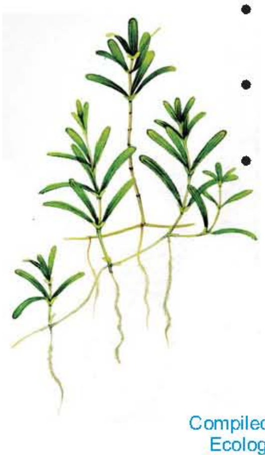

- Leaves with 3 veins
- 2-3 leaves at each node
- Leaves "whorl" around stem
- Found at subtidal depths
- Erect shoots 8-18cm long

Hd *Halophila decipiens*

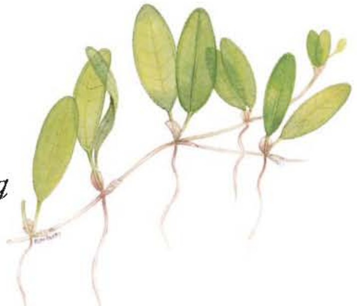

- Small oval leaf blade 1-2.5cm long
- 6-8 cross veins
- Leaf hairs on both sides
- Found at subtidal depths

Compiled by the Marine Plant Ecology Group, Northern Fisheries Centre CAIRNS, AUSTRALIA April 2002

Annex III – Sample small- and large-scale maps

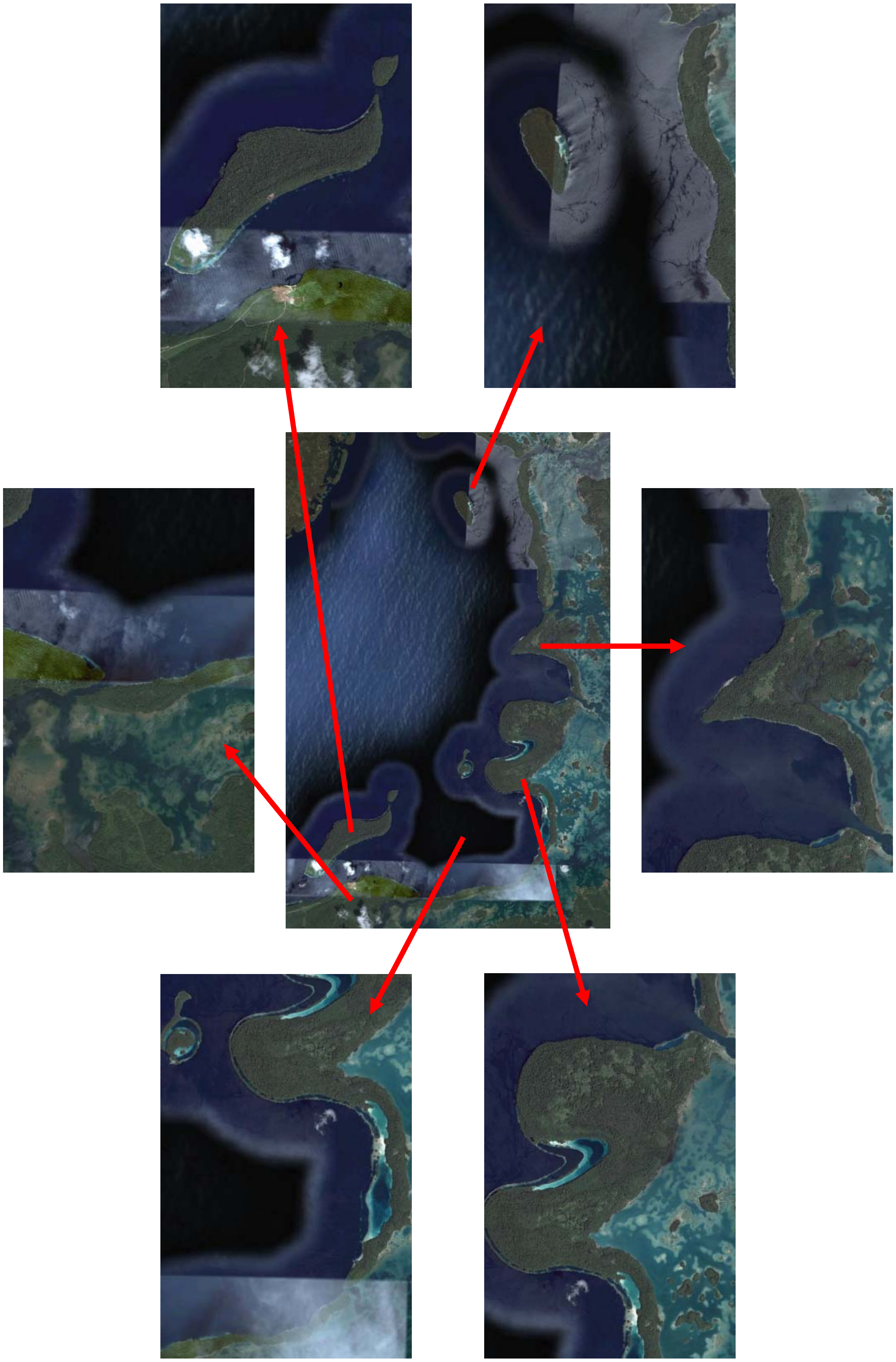

Supplement: S3 Appendix — (PDF) [file pone.0190021.s003.pdf]
